# On the Stability and Homogeneous Ensemble of Feature Selection for Predictive Maintenance: A Classification Application for Tool Condition Monitoring in Milling

**DOI:** 10.3390/s23094461

**Published:** 2023-05-03

**Authors:** Maryam Assafo, Jost Philipp Städter, Tenia Meisel, Peter Langendörfer

**Affiliations:** 1 Department of Wireless Systems, Brandenburg University of Technology Cottbus–Senftenberg, 03046 Cottbus, Germany; langendoerfer@ihp-microelectronics.com; 2 Department of Automation Technology, Brandenburg University of Technology Cottbus–Senftenberg, 03046 Cottbus, Germany; 3 Fraunhofer IPMS, 01109 Dresden, Germany; 4 IHP—Leibniz-Institut für innovative Mikroelektronik, 15236 Frankfurt, Germany

**Keywords:** classification, feature selection, homogeneous feature selection ensemble, predictive maintenance, milling, sensor fusion, stability of feature selection, tool condition monitoring

## Abstract

Feature selection (FS) represents an essential step for many machine learning-based predictive maintenance (PdM) applications, including various industrial processes, components, and monitoring tasks. The selected features not only serve as inputs to the learning models but also can influence further decisions and analysis, e.g., sensor selection and understandability of the PdM system. Hence, before deploying the PdM system, it is crucial to examine the reproducibility and robustness of the selected features under variations in the input data. This is particularly critical for real-world datasets with a low sample-to-dimension ratio (SDR). However, to the best of our knowledge, stability of the FS methods under data variations has not been considered yet in the field of PdM. This paper addresses this issue with an application to tool condition monitoring in milling, where classifiers based on support vector machines and random forest were employed. We used a five-fold cross-validation to evaluate three popular filter-based FS methods, namely Fisher score, minimum redundancy maximum relevance (mRMR), and ReliefF, in terms of both stability and macro-F1. Further, for each method, we investigated the impact of the homogeneous FS ensemble on both performance indicators. To gain broad insights, we used four (2:2) milling datasets obtained from our experiments and NASA’s repository, which differ in the operating conditions, sensors, SDR, number of classes, etc. For each dataset, the study was conducted for two individual sensors and their fusion. Among the conclusions: (1) Different FS methods can yield comparable macro-F1 yet considerably different FS stability values. (2) Fisher score (single and/or ensemble) is superior in most of the cases. (3) mRMR’s stability is overall the lowest, the most variable over different settings (e.g., sensor(s), subset cardinality), and the one that benefits the most from the ensemble.

## 1. Introduction

Maintaining the proper operation of industrial systems is crucial to meet the productivity and reliability requirements [1]. To this end, different maintenance strategies exist, among which the predictive maintenance (PdM) is particularly receiving a lot of interest from both industry and academia [2]. PdM is based on assessing the health condition of the monitored system so that a timely maintenance plan can be made accordingly. This is unlike other strategies where maintenance is performed only after the occurrence of failure (i.e., run-to-failure maintenance) or regularly based on a predefined schedule (i.e., preventive maintenance) [3]. PdM allows for preventing failures while maximizing the utilization of the industrial components as well as minimizing the downtime and maintenance costs [1].

The PdM mainly includes diagnosis tasks (e.g., fault identification, health state recognition) and prognosis tasks (e.g., prediction of the remaining useful life) [2]. The specific PdM application addressed in this paper is the classification-based tool condition monitoring (TCM) in milling, i.e., health diagnosis, where different data classes correspond to different health conditions of the tool. Milling is a popular machining process in which a rotating cutting tool is used to remove material from the workpiece in such a way that the desired geometry of the surface is attained. The tool, inevitably, wears gradually due to the joint effect of the heat and force generated during the milling process. The quality of the finished surface is directly affected by the tool wear. In addition, when the tool wear exceeds a certain level, a tool breakage occurs. Therefore, TCM is essential for ensuring good quality products and for replacing the tool in a timely manner [1,4].

**Figure 1:**
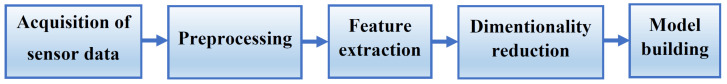
A general framework of the machine learning-based predictive maintenance in the offline phase.

The advances in collecting, storing, and processing large amounts of data are key enablers of data-driven PdM solutions [2,3,5]. Data-driven solutions include statistical methods, stochastic models, and machine learning (ML) methods. ML can perform complex tasks that might not be handled by other data-driven solutions. This is due to the ML advanced learning algorithms that can capture complex relations from data [2]. A general framework of ML-based PdM in the offline phase is depicted in Figure 1, and it encompasses the following steps [3,6]:

1. Acquiring raw sensor signals representing the process at hand, e.g., tool wear progression during machining.
2. Preprocessing the sensor signals, e.g., for reducing noise.
3. Extracting features from the sensor signals.
4. Reducing the data dimensionality (feature reduction), mainly through feature transformation or feature selection.
5. Building a learning model that can eventually be deployed in the online phase to assess the system health.

Feature extraction allows for reaching meaningful information about the underlying process. However, it is quite intricate, if not impossible, to theoretically determine the optimal sensory features for the problem at hand. This is mainly due to the complexity and dynamics of the industrial processes being monitored and the presence of diverse factors affecting the importance of a given feature, including the operating conditions [4,7,8], position of sensors, signal-to-noise ratio of the acquired signals [8], the window size of feature extraction [9], etc. Therefore, in order to ensure that sufficient information is derived, many features are initially extracted from the sensor signals in the time, frequency, and/or time-frequency domain. However, some of these features might be irrelevant and redundant [6], and the number of extracted features might be too large, causing the overfitting problem [8,10]. Hence, the role of the subsequent step, i.e., dimensionality reduction, is to provide a feature vector of a lower dimension while keeping the most representative information from the original features.

Dimensionality reduction is essential for model building as it enhances the generalizability of the model and reduces the storage and computation requirements. To this end, two major techniques exist, namely feature transformation and feature selection. In the former, the original feature space is mapped into another lower-dimensional space, and thus, completely new features are constructed, whereas in the latter, no transformation is performed, but rather, a subset of the most relevant features is selected from the entire feature set. Since feature selection preserves the physical meaning of the features, it outperforms the feature transformation with respect to the interpretability [11]. The focus of this paper is on feature selection (FS).

FS methods can be categorized based on the involvement of the learning algorithm into: filter, wrapper, and embedded methods.

- Filter methods function independently of any learning algorithm. They rely solely on the intrinsic properties of the features, e.g., their correlation with the output, variance, etc. Filter methods are characterized by being fast and computationally efficient, which is particularly beneficial for high-dimensional datasets. However, they usually lead to a lower classification accuracy compared to the other two categories [12].
- Wrapper methods utilize a specific learning method along with a search strategy that searches the space of possible feature subsets. Each drawn feature subset is evaluated based on the employed learning model’s performance, e.g., predictive accuracy, which is assessed using a validation set or cross-validation. As a result, wrappers suffer from the intensive computations needed, as each iteration involves training and testing the model from scratch [10]. Another disadvantage of wrappers is the risk of overfitting. Additionally, the selected features are specific to the learning algorithm employed [13].
- Embedded methods employ a learning algorithm that inherently assesses the feature importance as part of the model training, e.g., random forest. Therefore, these methods are computationally more efficient than the wrappers [12]. Similarly to wrappers, the embedded methods suffer from the bias of their output by the particular learning algorithm employed [13].

Only filter-based FS methods are considered in the experiments conducted in this paper, as they are less computationally expensive than the other two categories and also produce generic solutions due to their independence of the learning algorithm.

The output of a given FS method can take one of the three following forms, with *N* being the total number of the evaluated features [12]:

- Weights (scores), where weights are assigned to the *N* features based on their importance, as perceived by the employed FS method.
- Ranks, where the most important feature is usually ranked first, whereas the least important one is ranked *N*th.
- A subset of features selected from the original *N*-feature set.

Before training the model, a subset of selected features is eventually derived from the weights or ranks. The weights of features are sorted to produce a ranked list of features, and this list can be cut by specifying a specific number of features or a threshold (usually a percentage of the selected features) to generate a subset of the most important features [14].

FS methods have been extensively employed, investigated, and developed in the field of PdM, including a wide range of industrial processes, e.g., machining [4], industrial components, e.g., machining tools [4], gears [15], bearings [16], as well as PdM tasks, e.g., diagnosis [5] and prognosis [17]. Regarding the evaluation of FS methods, the majority of these works only consider the predictive power of the FS, expressed as the predictive performance of the learning model whose input are the features selected by the FS method, e.g., accuracy or similar measures [4]. Some works also consider other performance indicators such as the run time of the FS algorithm [18], among others. However, to the best of our knowledge, the stability of FS has not been considered in any of the works existing in the field of PdM. The stability of a given FS method reflects the ability of this method to select a consistent subset of features under small perturbations (variations) in the input data. Such data variations can be at the instance level and/or feature level [12]. The focus of this paper is on the instance-level data perturbations, i.e., when the variations are caused by adding or removing some data samples that come from the same process generating the data [12,19,20]. The stability of FS started to gain a lot of attention in many domain areas, especially where the data tend to have a high number of features but a small number of observations, e.g., in text mining and bioinformatics [12,21]. It has recently been addressed with various applications, e.g., sensor array optimization in electronic nose [22], and with different kinds of data, e.g., text, image, video, electrocardiogram (ECG) signal, voice recording [21]. However, it is still neglected in the area of PdM. Therefore, the main aim of this paper is to fill this research gap, both theoretically and experimentally. We argue that the stability of FS is an important aspect to be considered in the various PdM applications for many reasons, summarized as follows.

- The data used for PdM purposes are mostly time-series sensory signals that represent complex, dynamic industrial processes. These signals are usually non-stationary and contaminated with different kinds of noise. As such, estimating the stability of the FS outcome under changes in the dataset can reflect the extent to which the selected sensory features are generic and robust.
- The reproducibility of results is a crucial aspect to increase the confidence in the obtained outcome and the underlying implementation. Before the actual implementation of the PdM system, many elements should be determined in the offline phase, among which FS is of high significance as it selects the features that will be fed to the learning algorithm. Thus, the FS method that selects extremely different features for slightly different data samples may decrease the confidence in the total implementation, even if, in some cases, different subsets of features might build different models of equal accuracies.
- In the context of PdM, the selected features can be used by the domain experts to explore possible relations between some events, e.g., faults and health states, and the selected features. This is beneficial to reach a better understanding of the monitored system, which in turn can promote the PdM system. For such knowledge discovery and understandability purposes, stability of FS is of high importance.
- Many of the datasets acquired from real-world industrial processes contain only a small number of training observations. This is mainly due to the high requirements associated with acquiring large datasets in terms of cost, storage, and time [23]. Moreover, multisensor fusion is increasingly employed for PdM, leading to a larger pool of candidate features compared to the case of a single sensor. As such, it is not unlikely for the PdM dataset to suffer from a low sample-to-dimension ratio (SDR) (i.e., the ratio between the number of observations and number of features), in which case examining the FS stability would be particularly crucial.
- It is common in the literature to assess the candidate sensors based on the relevance of the features extracted from their signals, as performed in [4,24,25]. Such an assessment is especially important when there is a constraint on the number of the sensors to be used. In addition, the selected features in the offline phase will determine which signal analysis to be used in the online phase, e.g., fast Fourier transform, wavelet transform, etc. Thus, the FS stability can also affect the confidence in selecting the sensor(s) and signal analysis methods.

When it comes to facing FS stability issues, the trend in the literature seems to be toward developing techniques that can improve the stability of any FS method rather than establishing new FS methods that are stable. This is to be expected since there are already multitude of well-established FS methods and also given the fact that there is no single FS that is considered the best for all the applications. A recent technique that has been shown to increase the FS stability is the FS homogeneous ensemble. It was first introduced in [12] and is based on combining the outputs of identical FS methods that were fed with different data samples taken from the same dataset. This technique can be applied on all the FS types and algorithms, and it has been studied in various areas, e.g., bioinformatics, biomedicine [12], text mining [21], etc. To the best of our knowledge, it has also not been studied yet in the field of PdM.

Stability and homogeneous ensemble of FS are two recent research areas that have been studied in many fields but not yet in the PdM. This paper aims to fill this research gap with an application to TCM in milling. Our contributions in this paper can be summarized as follows:

1. Presenting a brief review of the existing performance indicators of FS methods in the PdM field.
2. Introducing the stability of FS as an important performance indicator to be considered in the field of PdM, which is, to the best of our knowledge, still neglected in this research area. Our motivation was driven by the reasons described above.
3. Presenting a brief review of the existing measures of FS stability while highlighting their pros and cons with respect to this study.
4. Investigating the impact of incorporating the homogeneous FS ensemble into the general framework of PdM. We experimented with different ensemble sizes and examined the impact of the FS ensemble on both the FS stability and predictive performance. Hence, this paper also contributes to the research area of FS ensemble, by exploring the potential of this technique for PdM.
5. Conducting a performance comparison of three well-known, filter-based FS methods, namely Fisher score, maximum relevance minimum redundancy (mRMR), and ReliefF, in terms of both the stability and predictive performance. This comparison is performed for both the single and ensemble versions of these methods and for different numbers of selected features.
6. The experimental study was performed for individual sensors and sensor fusion.
7. As mentioned previously, the PdM application addressed in this paper is classification-based TCM in milling. We used four milling datasets obtained from two sources: (1) milling experiments we conducted and (2) NASA’s data repository [26]. Our data and NASA’s data were generated under different experimental setups (different machines, milling tools, sensors, data acquisition systems (DAS), operating conditions, etc.). Further, they differ in the main data characteristics in terms of the number of classes and observations. Our aim of using such diverse milling datasets is to conduct a comprehensive, non-biased study with respect to the experimental cases and data characteristics.

The remainder of this paper can be summarized as follows. In Section 2, related works are reviewed. In Section 3, the methodologies used in this paper are presented, including the FS methods, the FS stability and its measures, and the homogeneous FS ensemble. In Section 4, the overall FS scheme implemented for both the single and ensemble versions of FS methods is described. Section 5 contains the implementation details concerning the datasets, experimental setups, data processes, results, and discussion. Finally, in Section 6, the conclusion and future work are presented.

## 2. Related Works

FS represents an integral part of many of the PdM frameworks existing in the literature. In many of these frameworks, traditional FS methods that were originally developed in the area of ML have been employed and investigated. For example, Fisher’s discriminant ratio [27], *t*-test [7], genetic algorithm [28], recursive feature elimination [29], and random forest [24] were used for TCM in different machining processes. In [30], mRMR was employed for machinery fault diagnosis. In addition, a performance comparison of different FS methods was conducted in some works. In [16], a comparison between sequential forward selection, sequential floating forward selection, and genetic algorithm was performed for fault diagnosis of bearing. In [17], three metaheuristic optimization FSs, namely Dragonfly, Harris hawk, and genetic algorithms, were compared for tool wear prediction. In [31], FS techniques based on decision trees, neural fuzzy systems, scatter matrix, and a cross-correlation were compared for TCM in milling. Aside from the well-known FS methods, some application-specific FS schemes were proposed in the field of PdM to meet particular needs and scenarios, e.g., an FS scheme was proposed in [4] to tackle the challenges related to anomaly detection of milling tools when the data belong to different operating conditions. In [5], an FS scheme was proposed to reduce the detrimental effect of the data outliers on the accuracy of fault diagnosis. Other general FS techniques were also developed in the PdM area with the aim of improving the diagnosis accuracy, as in [15,18].

To evaluate the effectiveness of the FS schemes proposed in the literature, their performance is usually compared with popular FS methods [4,15,25,32], with other related works [5], and/or with the case when no FS is performed (i.e., all the features are used) [5,8,15,25,33,34]. The major performance indicator used to evaluate a given FS or to compare different FSs is the predictive performance of the learning model, e.g., accuracy, which was built using the features selected by the corresponding FS method [4,15,16,17,18,25,32,33,34,35,36,37]. Other performance indicators of FS, not yet commonly used, include run time of the FS algorithm [18,34,37], training time of the learning model trained by the selected features [33,34], the computation complexity of FS in terms of the number of parameters to be adjusted in the FS algorithm [32], and the number of selected features [32,34,36]. However, as mentioned previously, to the best of our knowledge, stability of FS has not been considered in the field of PdM, including the various industrial processes, components, and monitoring tasks existing in this field. Table 1 summarizes the most common FS performance indicators used for PdM based on the existing literature.

A recent topic that has been studied in conjunction with the stability of FS is the homogeneous ensemble of FS [12,21,38,39,40]. It has been studied in many domain areas, mainly with the aim of improving the FS stability. Based on the existing works in the literature, there seems to be no general conclusion regarding the impact of homogeneous FS ensemble, as the ensemble performance is dependent on many factors, e.g., domain, dataset, FS method, aggregation method, etc. A broad study was conducted in [21], where the ensemble was tested in many domains, data sizes, and different FS types. It was found in [21] that the less stable the method is, the more it can benefit from the ensemble. Another common conclusion in the literature is that the homogeneous ensemble can increase the FS stability while maintaining (or even with slightly increasing) the classification performance [12,21]. However, there are examples where the FS homogeneous ensemble led to an improvement in the classification performance, but coupled with a decrease in the FS stability, e.g., the t-test ensemble with the cancer diagnosis microarrays datasets tested in [39]. Other examples exist when the increased stability caused by the homogeneous ensemble comes at the expense of decreasing the classification performance, e.g., the ensemble of random forest caused a decrease in the classification accuracy by an average of 10% compared to the single version for the biomarker discovery datasets tested in [12]. To the best of our knowledge, the homogeneous ensemble of FS has also not been explored yet in the field of PdM.

Stability and homogeneous ensemble of FS are two recent research areas that have been studied in many domain areas but not in the PdM. This paper aims to fill this research gap with an application to TCM in milling.

## 3. Methodologies

### 3.1. The Feature Selection Methods Studied in This Paper

As mentioned in Section 1, three filter-based FS methods are studied in this paper, namely Fisher score, mRMR, and ReliefF. These filters are supervised, i.e., the target classes are fed to the FS algorithm along with the observations, where each observation is represented by the features $F_{f};f=1,\ldots ,N$. These methods assign weights (scores) to the features, which can be further converted into ranks, and a subset (see the forms of FS output explained in Section 1). These particular methods were selected in this paper due to the fact that they are well-known, widely-used FS methods and that they are distinct from each other in terms of the general characteristics, as shown in Table 2. More specifically,

- The metric used for feature assessment in Fisher score, mRMR, and ReliefF is statistical, information-based, and instance-based, respectively.
- Regarding whether or not the assessment of a given feature is influenced by the other features, Fisher score is univariate, i.e., each feature is assessed independently of the others, whereas both mRMR and ReliefF are multivariate, i.e., the inter-dependency among features is considered.
- Regarding feature redundancy, only mRMR, among the three studied FSs, considers the redundancy among features.

More details about these methods are described as follows.

**Fisher score**: It is a simple-yet-effective FS method [41]. Each feature is evaluated individually based on its discriminant ability, $W_{f}$, expressed as in (1) [42].

(1) $$W_{f}=\frac{\sum_{c=1}^{C}n_{c}{\left(\mu_{f}^{c}-\mu_{f}\right)}^{2}}{\sum_{c=1}^{C}n_{c}{\left(\sigma_{f}^{c}\right)}^{2}}$$

where $n_{c}$ is the number of observations in class *c*. The number of classes is *C*. $\mu_{f}^{c}$ and ${\left(\sigma_{f}^{c}\right)}^{2}$ are the mean and variance of samples in class *c* corresponding to the feature $F_{f}$, respectively. $\mu_{f}$ denotes the mean of all the samples of feature $F_{f}$. Fisher score reflects the feature’s ability to globally maximize the between-class scatter (the numerator in (1)) and minimize the within-class scatter (the denominator in (1)). The major disadvantage of the Fisher score is that the relations between the features are not considered [42].

**mRMR**: It uses two mutual information-based criteria to score the features, namely relevance and redundancy. The most important features are considered to be the ones that are highly relevant to the class while being most dissimilar to the other features. The score given to the feature $F_{f}$ represents the mutual information quotient ${\mathrm{MIQ}}_{f}$, as given in (2) [43].

(2) $${\mathrm{MIQ}}_{f}=\frac{I(F_{f},Y)}{\frac{1}{\left|S\right|}\sum_{F_{z}\in S}I\left(F_{f},F_{z}\right)}$$

where the numerator and denominator of (2) represent the relevance and redundancy of the feature $F_{f}$, respectively. $I(F_{f},Y)$ is the mutual information between the feature $F_{f}$ and the target class *Y*, whereas $I(F_{f},F_{z})$ represents the mutual information between the features $F_{f}$ and $F_{z}$. *S* is the feature set.

**ReliefF**: A major advantage of the feature assessment performed by ReliefF is that the local dependencies between features are harnessed without loss of the global view [44]. The basic idea is to evaluate the features based on the extent to which their values discern the closely spaced observations. This assessment is performed iteratively. In each iteration, an observation is randomly drawn from the observation space. The *k* nearest neighbors of this observation are determined from the same class as well as from each of the other classes. The score of each feature is updated based on the difference of the feature values between the concerned observation and the nearest neighbors. The score, $W_{f}$, decreases if the feature values are different within the same class and increases if the feature values differ for the neighbors of the other classes, as shown in (3).

(3) $$W_{f}=W_{f}-\frac{1}{mk}\sum_{j=1}^{k}{\mathrm{diff}}_{f}\left(R_{i},H_{j}\right)+\frac{1}{mk}\sum_{c\neq \mathrm{class}(R_{i})}\frac{P(c)}{1-P(\mathrm{class}\left(R_{i}\right))}\sum_{j=1}^{k}{\mathrm{diff}}_{f}\left(R_{i},M_{j}\right)$$

where *m* is the number of iterations. $R_{i}$ is the observation drawn in the iteration *i*. $H_{j}$ and $M_{j}$ are neighboring observations to $R_{i}$ from the same class ($\mathrm{class}(R{}_{i})$) and a different class, respectively. P(c) is the prior probability of the class *c*, and is usually determined from the training samples fed to the ReliefF algorithm. The Equation (3) corresponds to the case where all the *K* nearest neighbors have the same influence (as indicated by the factor (1/k)), which is the version used in our implementation [44]. ${\mathrm{diff}}_{f}\left(.,.\right)$ is the value difference of the feature $F_{f}$ between two observations, and is given by (4).

(4) $${\mathrm{diff}}_{f}\left(O_{i},O_{j}\right)=\frac{\left|O_{if}-O_{jf}\right|}{\max\left(F_{f}\right)-\min\left(F_{f}\right)}$$

where $O_{if}$ and $O_{jf}$ are the values of feature $F_{f}$ for the observations $O_{i}$ and $O_{j}$, respectively. $\max(F_{f})$ and $\min(F_{f})$ are the maximum and minimum values of the feature $F_{f}$, respectively.

Recall that the data classes in our work represent different health conditions of the milling tool, and the importance score given to a certain sensory feature will reflect its discriminative power for tool health diagnosis as perceived by the respective FS method.

### 3.2. Stability of Feature Selection and the Measure Adopted in This Paper

The stability of a given FS method reflects the sensitivity of its outcome to small perturbations (variations) in the input data [45]. As mentioned previously, this paper focuses on the data perturbations at the instance level. Thus, stability reflects the consistency of the selected features when different subsamples from the same data-generating process are input to the FS algorithm [19,20], i.e., it is indicative of the reproducibility of the selected features [21,46].

The stability of FS was a neglected issue and is considered a research area of recent interest [21]. Various measures have been proposed in the literature to quantify the FS stability, most of which are essentially similarity measures, e.g., Pearson, Jaccard measures [45]. The input to the stability measure is the FS’s different outputs generated under different data versions of the original dataset. To quantify the stability, a pairwise similarity is first computed for different outputs, and then the overall estimated stability would be the average of the resulting similarities [45]. Let *X* be a dataset with *M* instances (observations) and *N* features, *m* is the number of perturbed versions of *X*. To quantify stability under instance perturbations, the *m* versions are usually created by resampling techniques, such as bootstrapping or cross-validation [14]. The total stability ($S_{\mathrm{tot}}$) is given as in (5) [12].

(5) $$S_{\mathrm{tot}}=\frac{2}{m(m-1)}\sum_{i=1}^{m-1}\sum_{j=i+1}^{m}S\left(O_{i},O_{j}\right)$$

where $O_{i}$ and $O_{j}$ are the two outputs of FS corresponding to the *i*th and *j*th data versions, respectively. S(.,.) is the similarity value between $O_{i}$ and $O_{j}$ computed based on the stability measure used.

Different stability measures exist for different types of FS output (weights, ranks, or subset) [12,45,47]. Nevertheless, some measures can be used for many output forms, e.g., Pearson’s correlation coefficient [45]. The Pearson’s correlation coefficient and the Spearman rank correlation coefficient are widely used for feature weighting and feature ranking, respectively [12,14,47]. As for the feature subsets, a variety of measures exist in the literature, most of which are basically increasing functions of the cardinality of the pairwise intersections, i.e., the higher the number of overlapping features between the feature subsets, the higher the stability is [20,45]. Examples of such measures include Jaccard’s, Hamming’s, and Kuncheva’s measures [45]. A thorough review on different stability measures can be found in [47].

The FS methods studied in this paper produce feature weightings that can be further converted into ranks and feature subsets. Thus, all the aforementioned types of stability measures can be applied. However, different types of measures represent the stability from different perspectives, and thus, provide different information. Weighting-/ranking-based measures estimate the stability from a global point of view as they take the weights/ranks of all the features into account. On the other hand, subset-based measures focus only on the subset of selected features, giving a finer view concerning the most important features [14]. Thus, the selection of the type(s) of stability measure should be driven by what insights are sought for the respective study. Since the main purpose of FS in this paper is to select the features that will be input to the classifier, we only focus on the subset-based stability measures.

As mentioned previously, most of the existing subset-based measures mainly rely on finding the features that the compared subsets have in common. Some of these measures suffer from specific drawbacks that result in constraints and/or invalid interpretations of the estimated stability in certain scenarios. For example, some measures, e.g., Kuncheva’s measure, require the compared feature subsets to be of the same cardinality [45]. Clearly, this constraint might not be met by the wrapper FS methods, for example. Other measures do not consider the so-called “similarity by chance” (the case when the stability corresponding to a random feature selection is dependent on the number of selected features) [48]. The usual trend for such measures is that the larger the subsets of selected features, the more features they have in common, as is the case with the Jaccard’s measure [45]. However, such a higher feature overlap is not linked to a higher stability in feature selection, but rather to a higher “chance”. To account for this issue, corrections are usually added to the stability measure so that the stability is zero for a random feature selection regardless of the number of selected features [45]. In addition, most of the existing measures assume that the different features are independent of one another [46] and, thus, regard features with different identifiers, e.g., $F_{i},F_{j};i\neq j$, as different regardless of whether they are highly correlated or not [20]. This drawback might lead, in the presence of highly correlated features, to having a seemingly low estimated stability when different yet similar features exist across the different output feature subsets [20,46]. The existing stability measures that consider the similarity among features are few, and a review on them can be found in [48]. Hence, in order to have a reliable quantification of the FS stability, it is critical to select a suitable measure for the respective study. For example, the issues related to having subsets with different cardinalities are not relevant to our work since we deal with ranking-based FSs, i.e., we can control that the number of the eventually selected features is constant over all the compared subsets. Based on the properties of the existing stability measures in the literature, and driven by what we consider desirable properties for our work, we adopted the stability measure proposed in [20], called SMA, for the following main reasons:

- It takes into account the issue of “similarity by chance” that was described above.
- It takes into account the correlation between the features belonging to different subsets. Thus, two features of different identifiers are considered similar if the correlation between them is high (compared to a predefined correlation threshold). This property is significant for PdM applications where the features extracted from the sensor signals might be highly correlated.
- In the SMA measure [20], the correlation-based similarity between features is assessed based on the absolute value of inter-feature correlation, which is unlike other existing measures that take the direction of correlation into account, such as the nPOGR measure proposed in [46]. The absolute value is relevant to our work since it represents the correlation strength (irrespectively of whether the correlation direction is positive or negative), which is the aspect of interest when examining the redundancy among features [4].

The aforementioned reasons represent the main properties that motivated us to adopt the SMA measure in this paper. However, it is worth mentioning that this measure also has other properties that were not mentioned above, e.g., it does not require the compared feature subsets to be of the same cardinality, which makes it applicable with all the FS algorithms including those that immediately generate a feature subset whose size might not necessarily be the same across different input samples. More properties and proofs of this measure can be found in detail in [20,48]. The SMA stability measure is given in (6) [20].

(6) $$\mathrm{SMA}=\frac{2}{m(m-1)}\sum_{i=1}^{m-1}\sum_{j=i+1}^{m}\frac{\left|V_{i}\cap V_{j}\right|+\mathrm{Adj}\left(V_{i},V_{j}\right)-E\left[\left|V_{i}\cap V_{j}\right|+\mathrm{Adj}\left(V_{i},V_{j}\right)\right]}{\mathrm{UB}\left[\left|V_{i}\cap V_{j}\right|\right]-E\left[\left|V_{i}\cap V_{j}\right|+\mathrm{Adj}\left(V_{i},V_{j}\right)\right]}$$

where $\left|V_{i}\cap V_{j}\right|$ is the number of overlapping features between the two feature subsets $V_{i}$ and $V_{j}$. $\mathrm{UB}\left[\left|V_{i}\cap V_{j}\right|\right]$ denotes an upper bound for $\left|V_{i}\cap V_{j}\right|$ and is set in this paper to $\sqrt{\left|V_{i}\right|.\left|V_{j}\right|}$, as in [20]. $\mathrm{Adj}\left(V_{i},V_{j}\right)$ is the adjustment function added to the stability measure to account for similarity between features from different subsets. Four different variants of this adjustment were proposed and investigated in [20]. They differ in the function used, but are similar in terms of the theoretical properties and the experimentally-attained stability values, as shown in [20]. Therefore, as recommended in [20], we will use the variant with the shortest execution time, which is called ${\mathrm{Adj}}_{\mathrm{Count}}\left(.,.\right)$, and is calculated as in (7).

(7) $${\mathrm{Adj}}_{\mathrm{Count}}\left(V_{i},V_{j}\right)=\min\left\{A\left(V_{i},V_{j}\right),A\left(V_{j},V_{i}\right)\right\}$$

where $A\left(V_{i},V_{j}\right)$ is computed as in (8):

(8) $$A\left(V_{i},V_{j}\right)=\left|\left\{x\in \left(V_{i}\setminus V_{j}\right):\exists \;y\in \left(V_{j}\setminus V_{i}\right)\mathrm{with}\;\mathrm{similarity}\left(x,y\right)\geq \theta \right\}\right|$$

Hence, $A\left(V_{i},V_{j}\right)$ represents the number of features that are included in $V_{i}$ but not in $V_{j}$ and has a similarity exceeding the threshold θ with at least one feature included in $V_{j}$ but not in $V_{i}$. Similarity (.,.) is assessed in this paper based on the absolute value of Pearson’s correlation coefficient, as it will be shown in Section 5.5.

As for $E\left[.\right]$ in (6), it represents the expected value corresponding to a random feature selection. Since this value is data-dependent, there is no general equation to calculate it. However, it can be estimated by repeating the following Monte Carlo procedure *L* times: (1) Performing a random feature selection, with each feature having an equal probability, to generate two feature subsets whose cardinalities are the same as the corresponding subsets $V_{i}$ and $V_{j}$, respectively. (2) Calculating the corresponding score $\left|V_{i}\cap V_{j}\right|+\mathrm{Adj}\left(V_{i},V_{j}\right)$. Then, $E\left[.\right]$ would be the average of the *L* scores. In this paper, *L* is set to 10,000 as in [20,46]. Estimating $E\left[.\right]$ will be performed for each pair of subsets $V_{j},V_{j};i<j$.

### 3.3. Homogeneous Ensemble of Feature Selection

The rationale of FS ensemble is similar to that of the ensemble learning. Ensemble learning was originally developed to enhance the performance of classification and regression tasks by combining the outputs of many individual models [12]. The diversity of these models is a crucial aspect of ensemble learning, and it can be induced at the algorithm level and/or the data level [21]. The ensemble concept has gained an increasing interest in the area of FS, mainly with the aim of increasing the stability of FS methods and the predictive performance [49].

The FS ensemble consists of two main components: individual feature selectors, also referred to as base selectors [21,49], and an aggregator. Each base selector generates a single output based on the input data and the type of the base selector (the applied FS method). Then, the aggregator combines the individual outputs to form one final output of the ensemble [12]. Many methods exist for both the base selectors and aggregators. Based on how the diversity is formed in the ensemble, the ensemble has two main categories: homogeneous and heterogeneous ensembles. In the former, the base selectors are all of the same type. However, the input data fed to them represent different versions of the original training data. On the other hand, the base selectors in the heterogeneous ensemble use the same input data, but they apply different FS methods on that data [21,49]. In this paper, only homogeneous ensemble is considered as it was experimentally shown to improve the stability of FS in many works, e.g., [12,21].

Different components and parameters should be considered when designing the homogeneous FS ensemble. First, instance perturbations are generated to attain the diversity needed for this ensemble category. To this end, sampling techniques are usually used, e.g., cross-validation [50], bootstrapping [12,38]. In this paper, bootstrapping is used to create *B* bootstrap samples with each one having *T* instances drawn, with replacement, from the training set; *T* is the number of instances in the training set. Thus, the employed FS will be applied to each one of these *B* bootstrap samples, resulting in *B* different outputs. The ensemble size, i.e., *B*, is a crucial design parameter. Hence, different values of this parameter will be tested in this paper, as it will be shown in Section 5.6.2. The resultant outputs will be eventually combined by an aggregator to produce the final output. The common aggregators existing in the literature combine rankings or subsets [12,49]. Rank-based aggregators are used in this paper. There exist several rank-based aggregators that range in complexity from aggregators with very simple functions, e.g., mean, median, etc., to more complex ones, e.g., the aggregator proposed in [38] that assigns bootstrap-dependent weights to the individual ranks of each feature based on the test accuracy of a classifier trained on the respective bootstrap sample. However, the sophisticated aggregators are more computationally expensive, without necessarily increasing the FS stability or the predictive accuracy [21]. Therefore, we will use a simple aggregator with a mean-based function as it was shown to perform well in many studies [21]. As such, the final rank of a given feature will be the mean of its individual *B* ranks, i.e., the final rank of feature $F_{f}$ is ${\mathrm{Rank}}_{f}=\mathrm{mean}\left({\mathrm{rank}}_{f1},\ldots ,{\mathrm{rank}}_{fB}\right)$. The final feature ranking of the ensemble would be an ascendingly sorted list of the feature ranks; the lower the feature’s rank, the higher the feature’s importance.

## 4. The Feature Selection Scheme Implemented in This Paper

For each of the three FS methods explained in Section 3.1, two versions are implemented, namely the single version and the homogeneous ensemble version (as described in Section 3.3).

As mentioned previously, redundancy among features are not taken into account by both Fisher score and ReliefF. However, redundant features, even if relevant to the class, increase the data dimensionality without necessarily enhancing, or even degrading, the representation of the process at hand. It was experimentally shown in [4] that, for a constant number of 5 features, an increase in the assessment accuracy of the milling tool was gained by simply replacing more-important yet redundant features with less-important but non-redundant ones. Additionally, it was shown in [51] that a more accurate fault diagnosis was achieved when redundant features are removed from a specific feature subset. Similarly to [4,51,52], we applied the following iterative procedure to eliminate redundant features, in which redundancy among features was examined using the Pearson’s correlation coefficient: In the first iteration, the top ranked feature eliminates the less important features with which it exhibits a correlation whose absolute value exceeds a threshold. In the next iteration, the same process is performed after determining the top ranked feature among the features remaining from the previous iteration, and so on until no feature is remaining. The Pearson’s correlation coefficient (absolute value), $R_{ab}$, between two features is given as in (9).

(9) $$R_{ab}=\left|\frac{\sum_{s}\left(y_{s}-\bar{y}\right)\left(z_{s}-\bar{z}\right)}{\sqrt{\sum_{s}{\left(y_{s}-\bar{y}\right)}^{2}}\sqrt{\sum_{s}{\left(z_{s}-\bar{z}\right)}^{2}}}\right|$$

where $y_{s}$ and $z_{s}$ are the feature samples corresponding to the features $F_{a}$ and $F_{b}$, respectively. $\bar{y}$ and $\bar{z}$ are the mean values of feature samples for $F_{a}$ and $F_{b}$, respectively.

The range of $R_{ab}$ is [0, 1]. In this paper, the condition for eliminating redundant features is $R_{ab}>=\mathrm{Th}$. A threshold value that is too high might not effectively remove redundant features. On the other hand, a threshold value that is too low might also lead to removing significant features. A common trade-off choice of this threshold is 0.9, as in [52,53,54,55,56], which is also the Th value used in this paper. This step of eliminating redundant features will be implemented for each of the studied FS methods. It should be noted that, even for mRMR which takes the redundancy among features into account, this step will not affect the feature preference perceived by this method, since mRMR considers the more-relevant, dissimilar features more important than the less-relevant, redundant ones.

**Figure 2:**
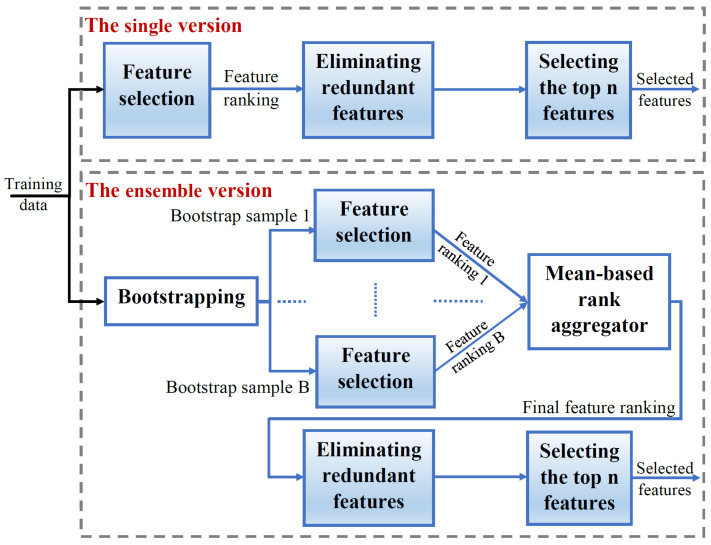
The overall feature selection scheme implemented in this paper for both the single and homogeneous ensemble versions of the FS method (Fisher score, mRMR, or ReliefF). The training set represented by the feature vectors is directly passed to the FS algorithm in the single version, whereas in the ensemble version, it undergoes the bootstrapping technique so that B different data versions are generated from the training set. The B bootstrap samples are fed to the same FS algorithm, resulting in B feature rankings. These rankings are combined using a mean-based aggregator to generate the final feature ranking of the ensemble. The next steps are the same as those applied in the single version, where redundant features are removed and the top n remaining features are selected.

Figure 2 illustrates the overall FS scheme implemented in this paper for both the single and ensemble versions of each FS method.

In the FS scheme, the training set represented by the feature vectors will be fed to the single version of a given FS method. However, for the ensemble version, the bootstrapping technique is applied on the training set to generate *B* bootstrap samples. Then, the respective FS method will be applied on each bootstrap sample to generate a corresponding feature ranking. As discussed in Section 3.3, we use a mean-based rank aggregator that averages the *B* individuals ranks generated for each feature. The resulting feature ranking will represent the ensemble feature ranking. For both the single and ensemble versions, the final feature ranking will be used to eliminate the redundant, less important features. Finally, the selected features will be the top *n* features among the remaining ones.

## 5. Implementation Details

This section presents all the details related to the datasets used, the workflow implemented, including preprocessing, feature extraction, FS (single and ensemble), and classification-based TCM, as well as the different evaluations performed, and finally the results and discussions. A five-fold cross-validation was used for the different evaluations performed in this section. The MATLAB R2020b software was used to perform the different experiments on the data.

### 5.1. Experimental Datasets

Four real milling datasets are used in this paper. Two of them, called DS1 and DS2 hereafter, are generated from our experiments, whereas the other two, called DS3 and DS4 hereafter, are from the NASA’s data repository [26]. Each of these four datasets contains run-to-failure data of the milling tool, i.e., the dataset covers the different stages of tool wear progression, starting from when the tool is completely new, through the different degradation states, and finally to the failure state. These datasets differ in many aspects, e.g., DAS, sensors, operating conditions, data size, etc. However, the main difference between the two datasets under each setup, i.e., (DS1, DS2) and (DS3, DS4), is the operating conditions of the milling process. The sensors and operating conditions of the four datasets are shown in Table 3. The detailed experimental setups will be described in the following two subsections.

#### 5.1.1. Experimental Setup and Preprocesses of Our Milling Datasets (DS1 and DS2)

**Figure 3:**
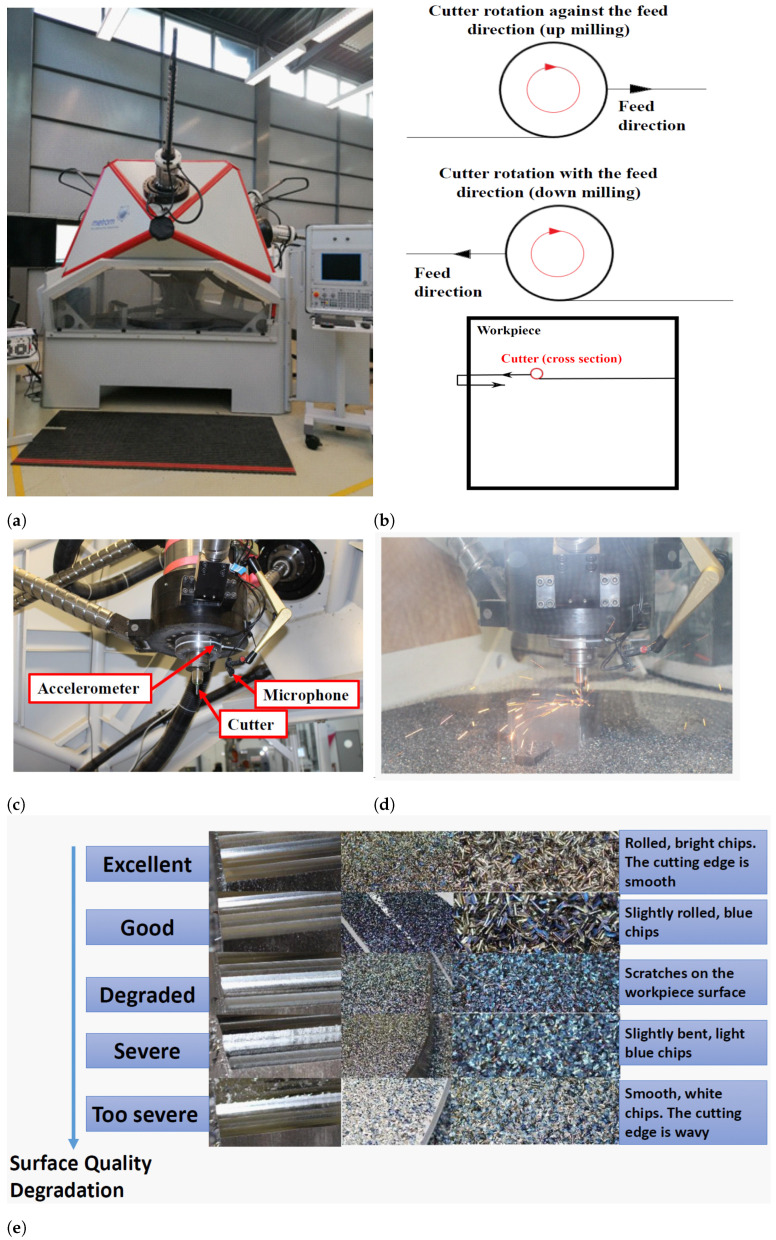
Our experimental setup. (**a**) CNC machine. (**b**) Up milling and down milling processes and the cutting paths on the workpiece. (**c**) The sensors and cutter inside the milling center. (**d**) Snapshot of the milling process. (**e**) The finished surface quality and the chips generated with the five classes of the tool health.

The milling machine used in our experiments is a five-axis milling center from Metrom GmbH that is equipped with an Andronic 2060 CNC control. We used solid carbide milling cutters with a diameter of 8 mm, cutting material of VHM, a tooth number of 2, and a cutting edge length/total length of 19 mm/63 mm. A workpiece of steel was used. The workpiece surface was machined line-by-line with the cutter. The milling performed is dry milling. For each collected run-to-failure dataset, the milling was performed under a distinct set of operating conditions defined mainly by the rotation speed, depth of cut, and feed rate (See Table 3). We used a radial depth of cut of 3.6 mm for all the experiments. Two types of milling processes were performed, namely up milling, in which the cutter rotates against the feed direction, and down milling, in which the cutter rotates along the feed direction. For each dataset, these two types were used alternately, i.e., the milling type changes with every cutting line. A microphone (model: SPU0410LR5H-QB) and an accelerometer (model: ADXL1005) were used to collect the sound and vibration signals, respectively. Five classes were used to label the data. To this end, several factors were taken into account for labeling, mainly the quality of the finished workpiece surface; the characteristics of the chips generated during milling, specifically, their color and shape; as well as the experience of the machine operator (also guided by the sound and vibration generated during milling). The first two factors, i.e., surface quality and chip characteristics, are largely influenced by the tool wear. As the tool wear increases, the surface quality degrades, the chip no longer rolls in as much, and the cutting temperature also increases due to the increased friction, which affects the chip color. Figure 3 illustrates the main setup of our milling experiments, including the CNC machine used, the sensors mounted inside the milling center, a snapshot of the milling process, the up and down milling processes, and the finished surface quality as well as the chips generated during milling corresponding to the five classes.

**Figure 4:**
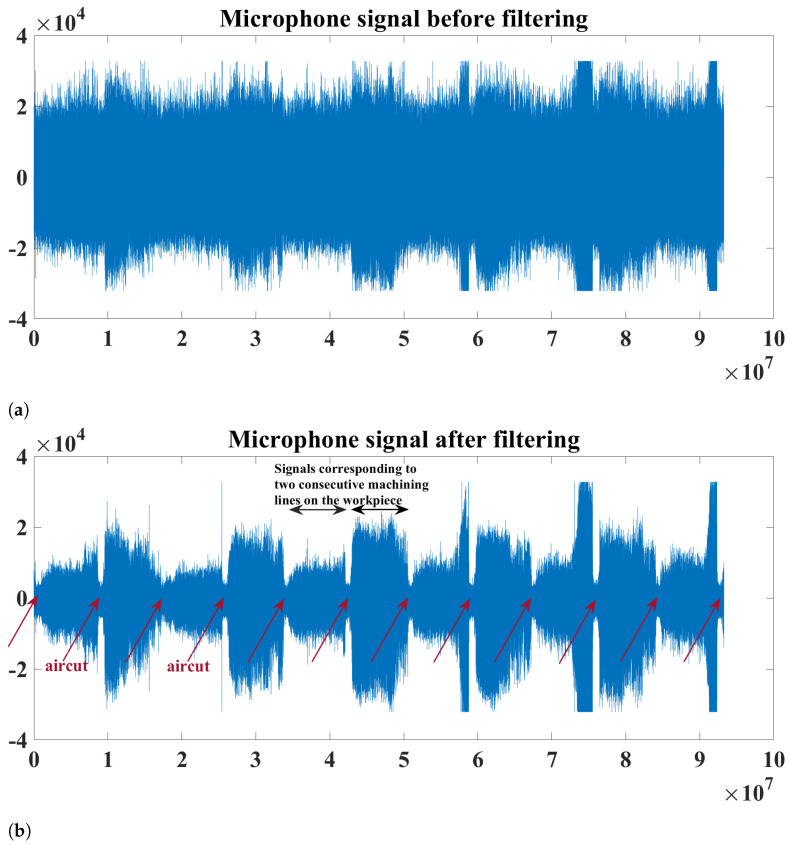
An example of the microphone signal during our milling experiments in the time domain: (**a**) before filtering and (**b**) after filtering. Some events, e.g., aircuts, cannot be recognized clearly before filtering.

The sensor signals were amplified by an amplifier and then fed to a Red Pitaya board that served as a data acquisition system in our setup. The sampling frequency for each of the sensors was about 1.95 MHz. The acquired sensor signals were then transferred to a PC where they were stored for further analysis. The signals of each sensor were stored in packages of 16,381 samples each, where the frequency of the packages is about 10 Hz. The following preprocesses were performed on each of the sensor signals: (1) Filtering using a median filter which was selected due to its capabilities of removing high-frequency noise without affecting the useful information. (2) As in [1,24], the parts of signals corresponding to the following events of the milling process were eliminated: the aircut (when the tool is in the air) and the entry/exit cuts which correspond to when the tool first engages/disengages in/out of the workpiece. (3) The original sampling frequency of 1.95 MHz was reduced to about 400 KHz, where the latter frequency, given the frequency ranges of the sensors used and the monitoring signals, is sufficient according to the Nyquist–Shannon sampling theorem which states that the sampling frequency should be at least twice as high as the maximum frequency contained in the sampled signal. In our case, the sampling frequency of 400 KHz is about 7 times higher than the maximum frequency of interest contained in the observed signals. Figure 4 illustrates an example of the microphone signal before filtering (Figure 4a) and after filtering (Figure 4b) for different machining lines. It can be noticed from the figure that, before filtering the signal, events such as when the tool is in the air cannot be clearly recognized. The signal parts corresponding to the aircuts are those parts whose amplitude is considerably lower than the other parts corresponding to the actual milling.

#### 5.1.2. Experimental Setup and Preprocesses of the NASA Milling Datasets

The publicly available NASA milling data [26] contains 16 run-to-failure datasets with 6 sensors. For this paper, we only used two datasets, namely case 9 and case 10. We only used two sensors, namely the AC current sensor of the spindle motor and the acoustic emission (AE) sensor mounted on the machining table. Each run of the machine is provided with sensor signals, with each signal containing 9000 samples obtained at a sampling frequency of 250 Hz. The data are labeled with a flank wear value (VB) that was measured after each run. As in [1], we divided the dataset into three classes based on their VB values as follows: VB < 0.2 mm, 0.2 mm ≤ VB ≤ 0.4 mm, and VB > 0.4 mm. We performed the following preprocesses on the NASA datasets: (1) Eliminating the aircut and entry/exit cuts, as described previously in our setup. (2) Segmenting the signal corresponding to the milling part of each run into 4 non-overlapping segments of 1024 samples each.

### 5.2. Feature Extraction

For feature extraction, we applied signal analysis methods that are quite similar to those used in [4]. The methods and the corresponding features are described as follows.

- Time-domain statistical analysis was used to calculate the following 8 statistical features: mean, variance, skewness, kurtosis, impulse factor, crest factor, root mean square (RMS), and range [4,6].
- A multi-resolution analysis was performed using a non-decimated discrete wavelet transform. For this, the following mother wavelet functions were used: db1 and db3. As in [4], with each of the mother wavelets, a six-level decomposition of the data segment was performed, resulting in six details (D1, D2, …, D6) and one approximation (A6). From each of these coefficients, the following statistical features were extracted: mean, variance, skewness, and kurtosis, constituting a total of 28 features. Since 2 mother wavelet functions were used, a total of 56 wavelet-based features were generated.
- Time-frequency analysis was performed to calculate the mean peak frequency. The mean peak frequency is the average of the peak frequencies determined at different time instances of the data segment, with each peak frequency being the frequency with the maximum power at the corresponding instance [4]. In [4], this feature was extracted from the scalogram generated by the continuous wavelet transform. However, in this paper, we extracted this feature from the spectrogram generated by the short-time Fourier transform (STFT) since this latter transform is less computationally expensive than the continuous wavelet transform.

**Figure 5:**
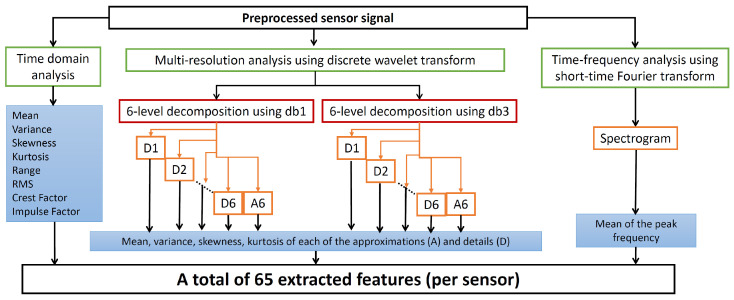
The methods used to extract sensory features in this paper.

As such, 65 (8 + 56 + 1) features were extracted out of each sensory segment, as depicted in Figure 5. After the feature extraction, each segment representing one observation, will be represented by the corresponding extracted features, rather than by raw sensor signals. As such, for an individual sensor, the 65 features corresponding to that sensor will be used for feature selection, whereas for the sensor fusion, the concatenated feature vectors from two sensors, i.e., a 130-feature vector, will be used. Table 4 shows the main characteristics of the four datasets used in this paper in terms of the number of classes, observations, and extracted features, as well as the SDR. SDR is a common measure used to reflect the difficulty of the task performed by the FS methods [12,21], with SDR << 1 being considered too challenging [21]. In this paper, SDR is only computed on the training set, since it is the data portion that the FS method will see. Since the same number of features were extracted for all the datasets, the SDR variation over the datasets is caused by merely the difference in the number of observations. As it can be noticed from the table, there is a considerable difference in SDR values between (DS1 and DS2) on the one hand, and (DS3 and DS4) on the other. For sensor fusion, for example, SDR for the datasets DS1–DS4 is 78.03, 96.40, 0.22, and 0.25, respectively. Such a huge difference allows for gaining diversified insights into the behavior of the FS methods and classifiers, as it will be shown later.

### 5.3. Feature Selection

Before performing the feature selection, the extracted features are normalized based on the training samples that will be input to the FS. For this purpose, the z-score is used in this paper as in (10).

(10) $$z_{jf}=\frac{x_{jf}-\bar{X_{f}}}{S_{f}}$$

where $x_{jf}$ and $z_{jf}$ are the original and normalized values of the sample *j* corresponding to the feature $F_{f}$. $\bar{X_{f}}$ and $S_{f}$ are the mean and standard deviation of the feature $F_{f}$’s samples, respectively.

As mentioned previously, Fisher score, mRMR, and ReliefF are applied for all the experiments performed in this paper. Regarding the parameters related to the implementation of ReliefF, the number of the nearest neighbors, *k* in (3), was set to 10. Additionally, all the observations input to the ReliefF algorithm were used for computing the feature weights, and all the *k* nearest neighbors contribute equally to the weight updates [44].

It is noteworthy that, for a given dataset, the Fisher score of a specific feature $F_{f}$ related to a specific sensor will be the same for both the cases of the individual sensor and the sensor fusion. This is unlike the scores given by mRMR and ReliefF, i.e., the scores given by these methods to the feature $F_{f}$ will vary between the single sensor and sensor fusion cases. This is due to the fact that the latter two methods are multivariate, and hence, scoring the feature $F_{f}$ will be influenced by the other input features.

### 5.4. Classification-Based Tool Condition Monitoring

As mentioned previously, TCM is modeled in this paper as a multi-class classification problem. We employed support vector machines (SVM) and random forest (RF) as classifiers for the following main reasons:

- SVM is one of the most popular classifiers that have shown a promising performance for TCM, e.g., in [1,24,28,29,33,37].
- RF is considered the most popular method of creating a decision forest—a model ensemble whose base learners are decision trees. It has been widely and satisfactorily used for diagnosis tasks of different industrial components, e.g., bearings [57], and it recently started gaining interest for TCM, e.g., in [58,59]. However, it is still not extensively investigated with different FS methods for classification-based TCM.

Recall that in this paper, a five-class classification was performed for DS1 and DS2, and a three-class classification for DS3 and DS4. The one-versus-one approach was employed to constitute the multi-class SVM-based classifier consisting of C(C−1)/2 binary classifiers, where *C* is the number of classes. Hence, the SVM classifier consists of 10 and 3 binary classifiers for the 5-class and 3-class classification problems, respectively. For each experimental case, we used the two following non-linear kernels: the polynomial kernel of order 2 (the quadratic kernel) and the radial basis function (RBF) kernel. The penalty parameter was fixed to 1 in all the experiments.

Regarding the RF implementation, the RF model consists of 50 classification trees, the minimal leaf size is set to 1, and the number of the randomly selected features for each decision split is the square root of the total number of input features.

### 5.5. Performance Indicators of FS Methods

As mentioned previously, the FS methods studied in this paper will be evaluated based on their stability and the predictive performance of the classifier trained with the features selected by the respective FS method. Given the classification-based TCM performed in this paper, the stability of a given FS method will reflect the sensitivity of the selected features, i.e., those considered by the method to be the most informative for distinguishing between the different health conditions, to variations in the training observations (drawn from the corresponding run-to-failure data). As discussed in Section 1, FS stability is a significant performance indicator to be considered prior the deployment of the PdM system. However, it should not solely be used to select the best FS method. Assessing the predictive power of the selected features is also critical to ensure that they allow building an accurate classification model that can eventually serve as the diagnosis model.

Regarding the stability, the SMA measure is adopted as discussed in Section 3.2. Recall that this measure considers two features $F_{a},F_{b}$ to be alike if they are either identical, i.e., a=b, or different, i.e., a≠b, but highly correlated in reference to a predefined correlation threshold. As mentioned previously, the similarity of two features in (8) was computed using the absolute value of the Pearson’s correlation coefficient, which is the same similarity measure we used to examine redundancy among features (See Section 4). θ in (8) was set to 0.9, which is also the same threshold we adopted to examine redundancy among features. As for evaluating the predictive performance, we will use the macro-F1 of the classifiers. Macro-F1 is calculated by averaging the individual F1-scores corresponding to the individual classes, as in (14) [60]. F1-score for class *c* is given in (13), and it represents the harmonic mean of the recall (given in (11)) and precision (given in (12)).

(11) $${\mathrm{Recall}}_{c}=\frac{\mathrm{TP}}{\mathrm{TP}+\mathrm{FN}}$$

(12) $${\mathrm{Precision}}_{c}=\frac{\mathrm{TP}}{\mathrm{TP}+\mathrm{FP}}$$

(13) $${\left(F1-\mathrm{score}\right)}_{c}=\frac{2\times {\mathrm{Recall}}_{c}\times {\mathrm{Precision}}_{c}}{{\mathrm{Recall}}_{c}+{\mathrm{Precision}}_{c}}$$

(14) $$\mathrm{macro}-F1=\frac{1}{C}\sum_{c=1}^{C}{\left(F1-\mathrm{score}\right)}_{c}$$

where TP and TN represent the number of test observations that were classified correctly as positive and negative, respectively. FP and FN represent the number of test observations that were wrongly classified as positive and negative, respectively. *C* is the number of classes.

A five-fold cross-validation was implemented. As such, for a given dataset, each of the five folds will be used once as the test set for the classifier, while the remaining four folds will constitute the training set used for the feature selection and training the classifier. For a given FS method (whether a single or ensemble version), the overall stability of FS and the macro-F1 of the classifier will be calculated as follows.

- The overall stability is estimated by the SMA measure, as in (6), where the input to this measure is the five feature subsets selected based on the five training sets. Thus, *m* in (6) is 5, and the computed 10 pairwise similarities are averaged to obtain the overall stability. It is noteworthy that, since we used 5-fold cross-validation, the percentage of overlapping observations for each pair of training sets is roughly 75%.
- The overall macro-F1 is calculated by averaging the five macro-F1 values corresponding to the five test sets, with each value being calculated as in (14).

### 5.6. Experimental Results and Discussions

In this section, many evaluations of the studied FS methods will be performed over all the datasets and sensor configurations and can be summarized as follows:

1. The macro-F1 of the FS single versions is evaluated over all the possible numbers of selected features. Based on the results, only specific subset cardinalities will be selected for the next experiments.
2. For each of the subset cardinalities specified in step 1, the stability of each FS method is evaluated for both its single and ensemble versions. Five ensemble sizes are tested for the ensemble versions. The ensemble size that achieves the highest stability will be determined for each experimental case to represent the corresponding ensemble version. Finally, a global stability profile will be presented for the single and ensemble versions for all the datasets.
3. The FS single and ensemble versions are evaluated in terms of the stability and macro-F1 for the subset cardinalities specified in step 1 and the ensemble sizes being as determined in step 2.
4. Based on the comprehensive results of step 3, we specify only one cardinality of feature subset for each combination of dataset/sensor(s), where the FS single and ensemble versions will be evaluated based on the harmonic mean of the stability and macro-F1. Based on the results, the superior FS method (in its superior version, i.e., single or ensemble) will be determined for each combination.

#### 5.6.1. Evaluation of FS Methods (Only Single Versions) in Terms of Macro-F1 over All the Possible Numbers of Selected Features

As mentioned previously, feature rankings can be generated from the FS methods studied in this paper. Thus, to generate a feature subset out of each ranking, a threshold or a specific number of features, *n*, should be determined to select the top features. The optimum number of selected features, with respect to the predictive performance, is influenced by many factors, including the number of training observations, the complexity of the dataset and the task at hand [4], the FS method [21], etc. In light of that, we first conducted exhaustive experiments in which a classification model was built and tested for each of all the possible feature subsets consisting of the top *n* features, starting from n=1 and up to the total number of features remaining after eliminating redundant features. Our aim of these experiments is to reveal the trend of the macro-F1 variation over the number of selected features, and accordingly, select only some cardinalities of interest for the further experiments. Moreover, such experiments allow for comparing the number of features required by the different FS methods to achieve the highest predictive performance for a specific classifier, as it will be shown later.

**Figure 6:**
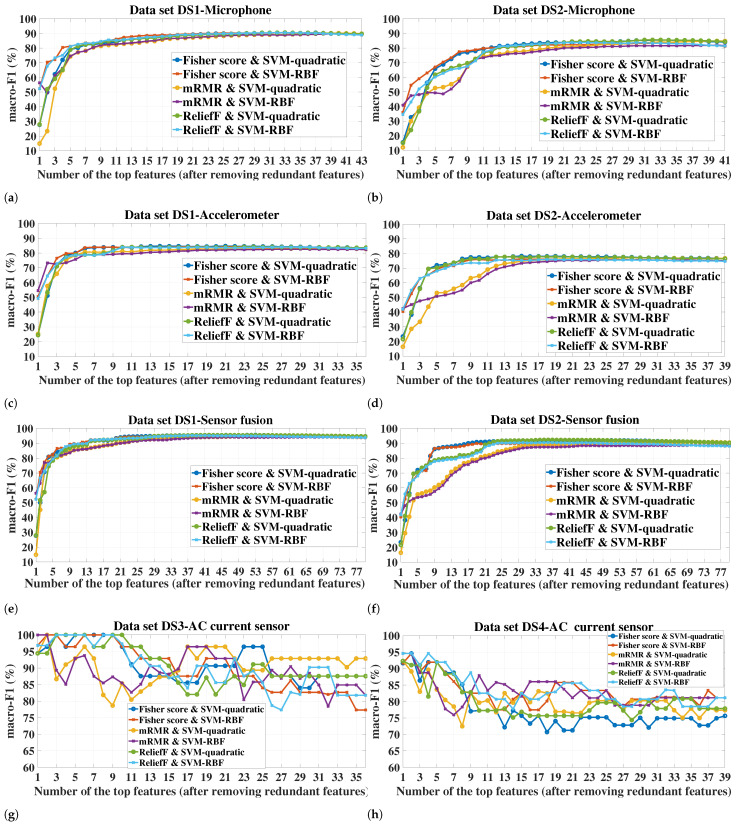

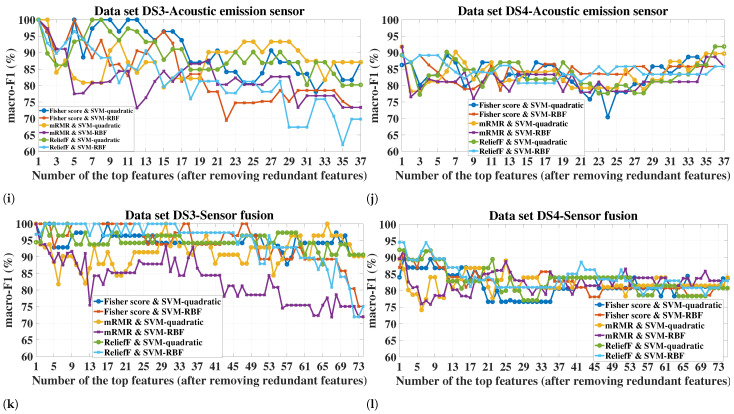
Macro-F1 (the average using 5-fold cross-validation) of the SVM classifiers as a function of the number of the top ranked features selected after removing the redundant features for different sensor configurations. (**a**–**f**) Datasets DS1 and DS2, (**g**–**l**) Datasets DS3 and DS4. The top features are added incrementally (one feature at a time), and a corresponding classification model is built and tested for each resulting feature subset. The aim of these experiments is to reveal the trend of the macro-F1 variation over the different cardinalities of feature subset, which in turn helps to select some cardianlities of interest for the further experiments and to show the number of features required by each FS method to achieve the highest performance for a specific classifier. For DS1 and DS2, adding more features improves the generalizability of the classification models until a certain number of features is reached, where the performance converges thereafter. The trend for DS3 and DS4 is different, as the macro-F1 reaches its peak value with only a few features (mostly 1–3 features) and thereafter fluctuates by adding further features with an overall decreasing trend. This can be attributed to the fact that the number of training observations for DS3 and DS4 is low, which increases the risk of overfitting with adding more features. The highest macro-F1 achieved for each case is given in Table 5.

Figure 6 shows the macro-F1 variation of the SVM classifiers over the number of selected features across all the datasets and sensor configurations. It can be vividly seen that the trends of macro-F1 variation corresponding to the datasets DS1 and DS2 for all the sensor configurations are more or less similar. Additionally, the trends with DS3 and DS4 are quite alike. However, the trend with both DS1 and DS2 differs largely from that with DS3 and DS4. Regarding DS1 and DS2, the macro-F1 increases with the addition of features, indicating an improvement in the classification performance of the models with more features added until a certain number of features is reached, where the macro-F1 does not change or slightly degrades with adding further features thereafter. For DS3 and DS4, however, the trend tends to be more complex, as the macro-F1 reaches its peak value with only a few features (mostly 1–3 features) and thereafter fluctuates with adding further features, with an overall decreasing trend. This can be attributed to the fact that the number of training observations for DS3 and DS4 is low (see Table 4), and thus, the risk of overfitting increases with adding more features, which adversely affects the classification performance on the test data. Moreover, the limited training samples increase the sensitivity of the learning to the information presented by new features, which can explain the fluctuation.

**Figure 7:**
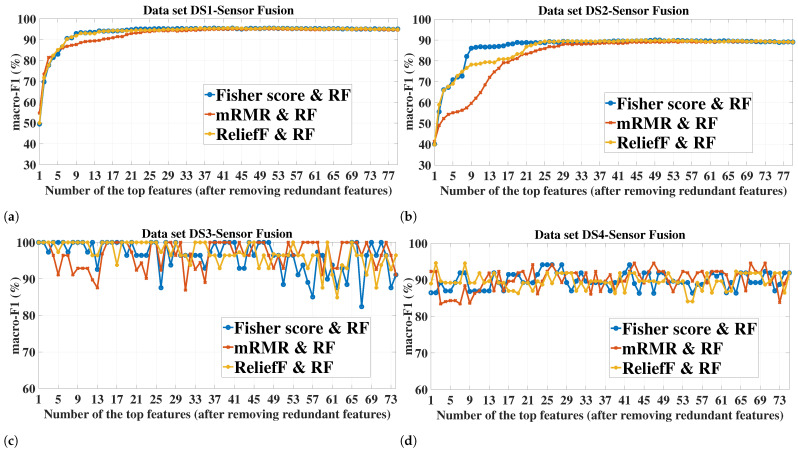
Macro-F1 (the average using 5-fold cross-validation) of the random forest classifier as a function of the number of the top ranked features selected after removing the redundant features, for the sensor fusion. (**a**–**d**) Datasets DS1–DS4. The highest macro-F1 achieved for each case is given in Table 5.

As it can be seen from Figure 7 corresponding to the sensor fusion case, the trend of the macro-F1 variation obtained with the RF classifier strongly resembles that obtained with the SVM classifiers for the respective dataset and FS method. The only significant difference in the trend is related to the small datasets DS3 and DS4 where RF showed a higher robustness to increasing the dimensionality compared to the SVM classifiers. For briefness, we only included the sensor fusion results in Figure 7 as the resemblance in the macro-F1 variation trend between the SVM and RF classifiers was also observed for the individual sensors.

For a given classifier, the number of features required to achieve a certain value of macro-F1 is dependent on the FS method. On the other hand, the superior FS method seems to be dependent on the number of features. For example, for all the sensor configurations in DS1 and DS2, mRMR led to the highest macro-F1 of both SVM-RBF and RF when only one feature was used. However, mRMR did not remain the superior when higher numbers of features were added (see Figure 6a–f and Figure 7a,b).

Regarding the performance comparison between the two SVM kernels, the superior kernel is largely dependent on the number of features. For example, for datasets DS1 and DS2, the RBF kernel tends to considerably outperform the quadratic kernel for all the FS methods when the number of features is low (around 4 features), e.g., for the case of the DS1-sensor fusion-one feature (Figure 6e), the difference in macro-F1 between the kernels reached 24.65%, 41.41%, 24.32%, for Fisher score, mRMR, and ReliefF, respectively. However, this difference diminishes rapidly with adding more features, and after a certain number of features, the quadratic kernel is mostly the superior of these two datasets.

Table 5 shows the maximum macro-F1 achieved by each FS method, along with the corresponding number of features. It should be noted that, for a given combination of dataset/sensor(s)-FS, the same macro-F1 value might be achieved with different numbers of selected features. For such cases, only the lowest number of features is recorded in Table 5. The table also shows the macro-F1 achieved when no FS was applied, i.e., all the features extracted from the sensor(s) were used to build and test the models. This latter case serves as a baseline for the FS methods to reflect the utility of applying the feature selection. An effective FS scheme should improve or at least not significantly degrade the classification performance compared to the case where no FS is applied. It can be seen from the table that this is fulfilled for all the datasets/sensor(s). In most of the experimental cases, the highest macro-F1 can be achieved with at least one of the three FS methods, which emphasizes the role of FS in improving the health assessment of the milling tool while simultaneously reducing the storage and computation requirements. This is especially true for the small datasets, such as DS3 and DS4, which increase the risk of overfitting when the feature dimension is relatively large. For example, all the FS methods in DS3-AC current achieved a macro-F1 of 100% with only 1–3 features for both SVM kernels, whereas all the 65 features (no FS) achieved 89.33% and 90.22% for the quadratic and RBF kernels, respectively. Concerning the cases where using all the features led to a higher classification performance compared to when FS was applied, the difference in the macro-F1 is mostly marginal and can be neglected given the higher number of features required, e.g., for the case corresponding to the accelerometer-quadratic kernel, a total of 65 features led to a macro-F1 of 85.65% and 78.31% for datasets DS1 and DS2, respectively, whereas for the same case, 84.74% and 78.27% were achieved by the Fisher score with only 21 and 15 features, respectively (i.e., a reduction by 44 and 50 features with a macro-F1 reduction by only 0.91% and 0.04%, respectively). For a given dataset, sensor(s), and classifier, the maximum achieved macro-F1 as well as the corresponding number of features are dependent on the FS method. It is always desirable to obtain a high predictive performance with the lowest number of features possible. For DS1 and DS2, the maximum F1-macro achieved by Fisher score and ReliefF is higher than that of mRMR for all the cases of the SVM classifiers and most of the cases of the RF classifier, and the number of required features is always lower than that of mRMR. However, mRMR with datasets DS3 and DS4 showed a comparable performance, and in some cases better (e.g., case of DS4-AE for both SVM-RBF and RF), compared to the other two FS methods.

Among the tested classifiers, RF achieved the highest macro-F1 with all the FS methods and sensor(s) for DS1, whereas SVM-quadratic was the superior for DS2. All the classifiers achieved a maximum macro-F1 value of 100% in all the cases of DS3, whereas for DS4, the superior classifier varies with the cases.

As for the sensor(s) used, the classification performance was better with sensor fusion compared to the individual sensors for DS1 and DS2. Taking SVM-quadratic as an example, the maximum macro-F1 that can be reached by sensor fusion is higher than that of the microphone by an average (over the three FS methods) of 4.93% and 6.5% for DS1 and DS2, respectively. The improvement gained by sensor fusion is even higher when compared to the accelerometer. However, for DS3 and DS4, the sensor fusion mostly led to an identical macro-F1 (and even lower with some cases of SVM) compared to the individual sensors. This represents an example of when adding more sensors might lead to more hardware and computation requirements without necessarily improving the performance.

Based on the macro-F1 values observed for the different datasets and sensor configurations in Figure 6 and Figure 7, and Table 5, the number of selected features, *n*, that will be considered for the next experiments is as follows:

- For datasets DS1 and DS2, we consider the value set of {10, 20, 30} for each of the individual sensors and {10, 20, 30, 40, 50} for the sensor fusion.
- For datasets DS3 and DS4, we consider the value set of {1, 2, 3} for each of the individual sensors and {1, 2, 3, 4, 5} for the sensor fusion.

#### 5.6.2. Stability of the FS Methods (Single Versions and Ensemble Versions with Different Sizes) for Different Numbers of Selected Features

**Figure 8:**
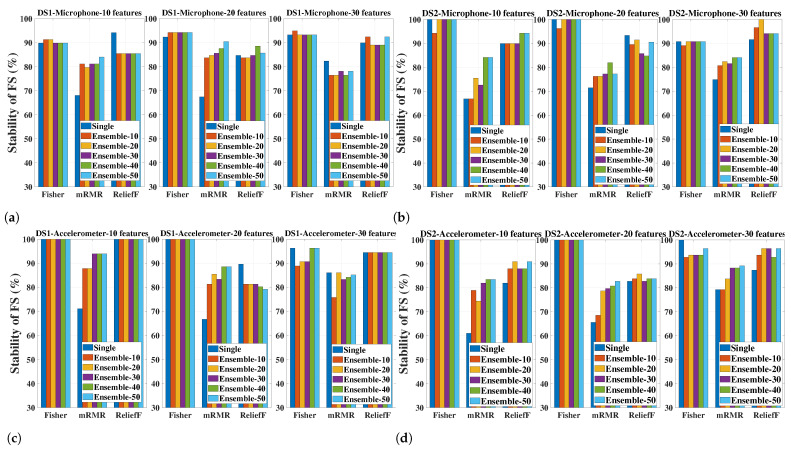

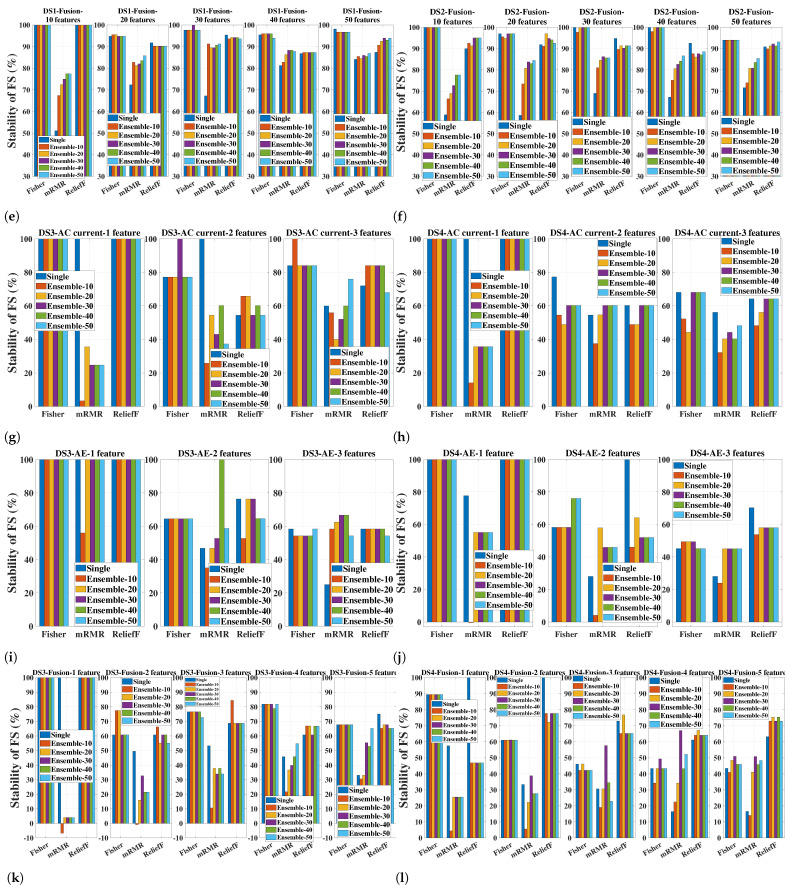
Stability (%) of the FS methods (single and ensemble versions) for different numbers of selected features and different sensor configurations. Different ensemble sizes (Ensemble-B) are tested, where B is the number of bootstrap samples used to construct the homogeneous ensemble of each FS method. (**a**–**f**) Datasets DS1 and DS2, (**g**–**l**) Datasets DS3 and DS4.

In this subsection, the stability of the studied FS methods will be examined for the numbers of selected features specified in Section 5.6.1. This will be performed for both the single and ensemble versions of FS methods. For each fold of the five-fold cross-validation, the corresponding training set will be fed to the single version and ensemble versions of a given FS method as illustrated in Figure 2. Recall that *B* is the number of bootstrap samples (the ensemble size). *B* is a significant design parameter of the ensemble. The experiments conducted in many different domains in [21] showed that a value of 50 for *B* is sufficient to increase the FS stability, and that higher numbers led to little or no improvement. Similarly, 30 and 40 bootstrap samples were shown to be sufficient in [12,39], respectively. Hence, in this paper, we will not consider more than 50 bootstrap samples. We tested five *B* values: 10, 20, 30, 40, and 50. Figure 8 shows the stability of the studied FS methods for both the single versions as well as the ensemble versions with different ensemble sizes for different feature subsets cardinalities. The following can be noticed from Figure 8:

- Different FS methods can largely differ in their stability, e.g., in the case of DS2-sensor fusion-20 features, the stability values of the single versions of Fisher score, mRMR, and ReliefF are 97.05%, 58.69%, and 91.88%, respectively, i.e., a noticeable difference between the Fisher score and ReliefF on the one hand, and mRMR on the other hand. Such a difference reflects the interaction between the data and the inherent functioning of the different FS methods and it promotes the role of the stability as a decisive performance indicator when it comes to selecting the best FS method for a PdM task.
- Concerning the FS single versions, mRMR has the lowest stability in all the datasets and sensor configurations, except for some feature cardinalities related to cases of DS3 and DS4, where it showed a similar stability to the other two methods (see Figure 8g–h).
- Concerning the FS ensemble versions, the stability of mRMR is the one most influenced by the ensemble technique. This manifests itself in the stability variation over the single version and the ensemble versions of the different sizes. On the other hand, the stability of Fisher score is the least influenced by the ensemble.
- Applying the ensemble technique can improve the stability of a given FS method, especially with mRMR in DS1 and DS2. However, there are also cases where the stability dropped with the ensemble, such as for mRMR in the case of DS4-AC current sensor-1 feature (see Figure 8h) and ReliefF in the case of DS1-Microphone-10 features (see Figure 8a).
- The stability can significantly vary over different ensemble sizes, as mostly seen with mRMR, e.g., in the case of DS4-sensor fusion-4 features, the mRMR ensemble with 10 and 30 bootstrap samples improves the stability over that of the single version by 5.98% and 50.69%, respectively. This emphasizes the importance of selecting the ensemble size experimentally so that the most benefit can be obtained when there is an improvement potential with the ensemble.
- The only negative stability values were obtained by the mRMR ensemble: −6.74% and −0.84% in the case of DS3-sensor fusion-10 bootstrap samples for one and two features, respectively (see Figure 8k), and −0.38% in the case of DS4-AE-10 bootstraps-1 feature (see Figure 8j). Obtaining a negative value for the adopted stability measure indicates that the stability is worse than that of a random feature selection for the corresponding case.
- Generally speaking, the stability of a given FS (whether single or ensemble) is influenced by the dataset, sensor(s), and the number of selected features.

For the next experiments and analysis, only the ensemble size that achieved the highest stability will be considered for the corresponding experimental case. The best ensemble sizes based on Figure 8 are given in Table 6. When the highest stability is achieved by different ensemble sizes, the largest ensemble size is recorded in the table since it will be more representative of the ensemble version.

**Figure 9:**
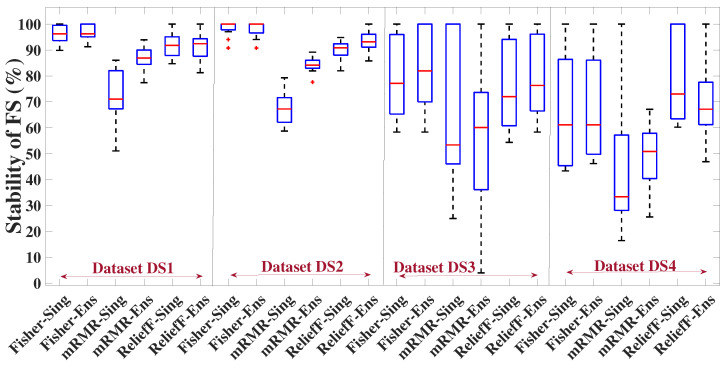
Distribution of stability values achieved by the single and ensemble FS versions over the different sensor configurations and subset cardinalities for each dataset. Fisher score was the most stable method for DS1–DS3, whereas ReliefF was the superior for DS4. The stability of mRMR is the lowest, the most variable, and the one that benefits the most from the ensemble technique.

In order to provide a global stability profile of the FS methods, revealing their overall stability performance for each dataset as well as the extent to which their stability can vary with different settings, Figure 9 shows a statistical summary represented by a box plot of the stability values achieved by the respective method across the different sensor configurations and subset cardinalities studied for the corresponding dataset. In each boxplot, the horizontal, red line represents the median. Additionally, the longer the box plot and the whiskers, the larger the stability variability, and vice versa. Recall that, as far as the sample size is concerned, DS1 and DS2 do not pose a challenge to the FS stability, as opposed to DS3 and DS4, which have very small training samples (see Table 4). Starting with DS1 and DS2, it can be seen that Fisher score (both single and ensemble) achieved the highest overall median stability (96.27% and 100% for DS1 and DS2, respectively) and showed the most consistent stability performance over the different sensor(s) and numbers of features studied under each dataset, which increases the confidence in considering the Fisher score the most globally stable method for these two datasets irrespectively of the underlying settings. On the other hand, mRMR showed clearly the lowest stability as well as the largest stability variability over the different cases for DS1 and DS2. This indicates that its stability in selecting the features relevant to TCM can vary largely with different sensor(s) and numbers of features for the same dataset. However, applying the ensemble technique significantly improved the overall stability of mRMR and also reduced its stability variability. Moreover, since the overall stability of Fisher score and ReliefF was already high in their single versions and did not get influenced much by the ensemble, the stability difference between the mRMR ensemble and those of the other two methods diminished noticeably, making mRMR more competitive under its ensemble version. As for DS3 and DS4, Fisher score and ReliefF showed the highest median stability, respectively. However, the stability variability of all the FS methods is larger compared to DS1 and DS2, especially mRMR, showing a larger dependence on the specific settings. This holds true even when the ensemble brought improvements. Further, a large overlapping between the stability values of the different methods can be observed. This all indicates that there is no global winner with respect to stability for these two datasets, and the best method should be determined based on the specific case. The behavior of the FS methods observed with these two datasets can be largely attributed to the small data size.

Figure 9 is meaningful to show the overall stability behavior of the different FS methods. However, when it comes to selecting the best FS method for a PdM system, a case-specific evaluation should be considered, i.e., with respect to the specific dataset, sensor(s) and number of features used in the PdM system. Moreover, as mentioned previously, stability should not be considered solely for evaluating the FS methods but along with the predictive power. This is what will be shown in the next subsection.

#### 5.6.3. Comparison of the Studied FS Methods (Single and Ensemble Versions) in Terms of Both Stability and Macro-F1 for Different Numbers of Selected Features

Table 7 and Table 8 show the results of stability and macro-F1 of the FS methods for the datasets (DS1 and DS2) and (DS3 and DS4), respectively. Among the single (or ensemble) versions of the FS methods, the bold value, for a specific performance indicator, indicates the superior method for the corresponding combination of dataset, sensor configuration, classifier, and feature subset cardinality, whereas the underlined values indicate which of the single and ensemble versions of a specific FS is better for the corresponding case. Some general observations based on these two tables are that, for a specific performance indicator, there is no single FS method that is superior for all the cases. Moreover, the superior FS method might vary depending on whether the single or ensemble FS versions are compared, owing to the varying influence of the ensemble technique on the different FS methods for the same experimental case. Similarly, regarding the performance of given FS method, neither the single version nor the ensemble is always superior.

Starting with the single FS versions in Table 7, Fisher score was the most stable FS method in almost all the sensor configurations and subset cardinalities for DS1 and DS2. More specifically, it was the superior for the cases related to the accelerometer (6 out of 6 cases), sensor fusion (10 out of 10 cases), and microphone (4 out of 6 cases). Indeed, the stability of Fisher score reached 100% in many cases, which is the upper bound value for the adopted stability measure. As for macro-F1, Fisher score was also mostly the superior, whereas mRMR was overall ranked the last with respect to the two performance indicators. The performance difference between the FS methods tends to be more noticeable with regard to the stability than to the macro-F1. For example, in the case of DS1-sensor fusion-10 features, the stability of both Fisher score and ReliefF was 100%, while it was 51.06% for mRMR (a stability difference of 48.94%). However, for the same case, the macro-F1 difference between mRMR and the other two FS methods did not exceed 3.44%, 4.51%, and 4.79% for the SVM-quadratic, SVM-RBF, and RF, respectively. Moreover, for a specific classifier, the difference in macro-F1 between the different FS methods is more significant when the number of features is lower, which reflects the difference in the predictive power of the top features selected by each method.

For DS1, all the single FS versions achieved a higher FS stability (or comparable) with the accelerometer than with the microphone. However, for DS2, the ReliefF stability with the microphone was always higher than that obtained with the accelerometer, e.g., by 10.57% when 20 features were used. As for mRMR in DS2, the superior sensor with respect to the stability of its features is dependent on the feature cardinality. For a given FS method, the stability variation over different sensors can indicate the interaction between the functioning of the FS method on the one hand and the robustness of the sensor’s signals and its features on the other hand. Compared to the stability with the individual sensors, the stability of FS methods with the sensor fusion is higher or lower or somewhere in between. However, regarding macro-F1, sensor fusion is always better than using one sensor alone for DS1 and DS2.

As for the impact of the ensemble, it can be noticed from Table 7 that the stability with the ensemble version is mostly higher or comparable to that of the single version, with few exceptions, e.g., the case DS1-accelerometer-20 features where the ReliefF stability dropped by 8.32% with the ensemble. It can be noticed that mRMR is the method most benefiting from the ensemble in terms of stability. The stability improvement with the mRMR ensemble reached as high as 26.35% (in DS1-sensor fusion-10 features). The stability of Fisher score is the least affected by applying the ensemble, and overall it remained the same. The impact of the ensemble on the macro-F1 also varies with different FS methods. The ensemble version led to more or less the same macro-F1 as that of the single version in all the cases of DS1 and DS2 with Fisher score and DS1 with ReliefF. However, there are cases with ReliefF and mRMR in which the stability improvement brought by the ensemble was at the expense of the macro-F1, as in the case of DS2-sensor fusion-30 features for mRMR (a macro-F1 degradation by 5.2%, 4.48%, and 5.83% for the quadratic SVM, RBF SVM, and RF, respectively) and other cases where the ensemble achieved an improvement in both the stability and macro-F1, e.g., for ReliefF in DS2-sensor fusion-20 features, where the ensemble increased the stability by 5.17% and the macro-F1 by around 3% for both SVM kernels and by 2.32% for RF and for mRMR in DS2-sensor fusion-10 features, where the ensemble increased the stability by 18.67% and the macro-F1 by an average of 17.64% over all the classifiers. The biggest macro-F1 improvement/degradation brought by the ensemble reached 3.25%/2.71% for ReliefF and 18.92%/6.53% for mRMR. Overall, the biggest ensemble impact was seen with mRMR.

Thus, for DS1 and DS2, Fisher score (both single and ensemble versions) was almost always superior in terms of both the stability and predictive performance. Even in the few cases where it was not superior, it showed a high and comparable performance. Hence, it can be considered globally the best method to select stable and predictive features for TCM with these datasets.

As for Table 8 showing the results for the datasets DS3 and DS4 which have small training samples, it can also be seen that different FS methods can differ significantly in terms of stability. This is especially apparent with the AE and sensor fusion of DS4 where the stability of ReliefF was considerably higher than that of the other methods, e.g., in DS4-AE-2 features, the stability of ReliefF single version is 100% whereas that of Fisher score and mRMR is 58.24% and 28.08%, respectively. While the features selected by all the FS methods were powerful enough to achieve a high diagnosis performance with these two datasets, their stabilities suffered in some cases. This indicates that the small size can have a more negative impact on the FS stability than on the model generalizability. Similarly to DS1 and DS2, the single version of mRMR with DS3 and DS4 mostly ranks the last among the three methods in terms of stability. The only case where the mRMR stability dominated that of the other two FS methods, including DS1 and DS2, is the case DS3-AC current-2 features in which the mRMR stability was 100%, while the stability of Fisher score and ReliefF were 77.14% and 54.35%, respectively. The single versions of Fisher score and mRMR achieved with the AC current sensor an identical or higher stability compared to the AE sensor. As for ReliefF, its stability tends to be higher with the AE sensor. Among the numbers of features tested for DS3 and DS4, all the three FS methods seem to be more stable when only the top feature is selected.

Regarding the ensemble versions, the stability of Fisher score is more influenced by the ensemble technique with DS3 and DS4 compared to DS1 and DS2. This can be largely linked to the statistical nature of the Fisher score that benefits from the large sample size of DS1 and DS2, which enabled the Fisher score to have a high stability, both in its single and ensemble versions. The ensemble of Fisher score maintained or improved the stability and macro-F1 in all the cases of DS3. The same holds true for ReliefF, with the exception being DS3-sensor fusion-5 features where the ensemble degrades the performance of ReliefF. Additionally, the ensemble degraded the stability of ReliefF with some cases in DS4, i.e., for 2–3 features of AE and 1–2 features of sensor fusion, where the stability of the single version was 100% in three of them. As for mRMR, when only one or two features are selected, the ensemble had a detrimental impact on mRMR in terms of stability and/or macro-F1 for almost all the sensor configurations concerning DS3 and DS4. However, for some cases of higher numbers of features, the ensemble can still be beneficial to mRMR, e.g., in the case DS4-sensor fusion-4 features, the ensemble increased the mRMR stability by 50.69% and the macro-F1 of SVM-quadratic, SVM-RBF, and RF by 13.02%, 8.11%, and 7.89%, respectively. Indeed, for 4–5 features of DS4-sensor fusion, the ensemble was mostly beneficial to all the FS methods in terms of both stability and macro-F1. It can be noticed that, for some cases, the ensemble allows for a more solid choice of the best FS method given a specific classifier. For example, in DS3-sensor fusion-3 features, both the single and ensemble versions of Fisher score and ReliefF yielded a macro-F1 of 100% for both SVM kernels. Regarding stability for the same case, the single version of Fisher score was the best with a stability of 76.67%. However, the stability of ReliefF ensemble (84.42%) exceeds all the other stabilities of both the single and ensemble versions of the other methods. Clearly, the ReliefF ensemble would be superior for this case with respect to both stability and macro-F1.

Recall that the main difference between the datasets under each pair of (DS1, DS2) and (DS3, DS4) is the operating conditions. The varying results over each two datasets are largely linked to the milling operating conditions that affect the sensory features.

#### 5.6.4. Comparison of the Studied FS Methods (Single and Ensemble Versions) in Terms of the Harmonic Mean of their Stability and Macro-F1

After studying the FS stability and predictive performance separately, it is worth having an overall expression that jointly reflects both performance indicators. To this end, we adopted the approach used in [12] in which the harmonic mean of the stability and classification performance is calculated. Given the performance indicators used in our paper, i.e., stability (SMA) and macro-F1, the harmonic mean (HM) is given as in (15), which corresponds to when both performance indicators are equally important.

(15) $$\mathrm{HM}=\frac{2\times \mathrm{SMA}\times \mathrm{macro}-F1}{\mathrm{SMA}+\mathrm{macro}-F1}$$

As it was shown in the previous experiments and analysis, the evaluation was performed for different numbers of features to gain broad insights. However, for the actual implementation of PdM system, a specific feature subset should be selected. Since the ultimate goal is to achieve an accurate tool condition monitoring, the feature subset cardinality at which all the FS methods led to a reasonable (maximum or nearly maximum) macro-F1 is selected for the corresponding case. Therefore, to simplify the final evaluation, we will select only one feature subset cardinality, *n*, for each pair of dataset/sensor configuration. For datasets DS1 and DS2, *n* was set to be 30, 20, and 40 for the microphone, accelerometer, and sensor fusion, respectively, whereas it was set to 2 for all the sensor configurations of DS3 and DS4. Even though a macro-F1 of 100% was achieved with only one feature in some cases of DS3 and DS4, we consider that using only one feature (regardless of the sensor) might limit the robustness of the monitoring system. Thus, we consider two features for these datasets.

Table 9 shows the HM results for the FS methods across all the datasets and for the subset cardinalities specified above. The superior FS method for each combination of dataset/sensor(s)-classifier, as well as the overall impact of the ensemble can also be found in this table. For all the sensor configurations of DS1, Fisher score (in its single and/or ensemble version) was the superior. The same holds true for DS2, with the exception being DS2-microphone where the ReliefF ensemble was the best. Both Fisher score and mRMR achieved an HM of 100% in DS3-AC current. ReliefF was superior for the AE sensors for both DS3 and DS4. Overall, based on all the four datasets, sensor(s), and classifiers, Fisher score exhibited the superior performance in most of the cases (24 out of 36 cases).

It is noteworthy that the methodology presented in this work to evaluate the FS methods, including the main experimental design, stability measure, ensembles, etc., can also be applied with other PdM applications, i.e., diagnosis tasks of other industrial components, prognosis tasks, etc., as well as other FS types, i.e., wrapper and embedded FS methods.

## 6. Conclusions and Future Works

Feature selection (FS) represents an integral part of many predictive maintenance frameworks. The main aim of this paper is to shed light on the significance of examining the stability of FS for the PdM applications and to investigate the potential of the FS homogeneous ensemble in this field. The FS stability reflects the extent to which the selected features are generic and robust for the monitoring task, and it influences the confidence in the implemented PdM system, including the built model and selected sensor(s), etc. The specific PdM application addressed in this paper is classification-based tool condition monitoring in milling. We used four milling datasets: two datasets generated from milling experiments that we conducted (called DS1 and DS2) as well as two datasets from NASA’s repository (called DS3 and DS4). These datasets differ in many aspects, including the milling machine, operating conditions, sensor(s), data acquisition system, sample-to-dimension ratios (SDR), number of classes, etc. Such diversified datasets helped to reveal the behavior of the FS methods with different data characteristics and experimental milling settings. We conducted a comprehensive performance comparison between three widely-used filter-based FS methods, namely Fisher score, mRMR, and ReliefF in terms of stability and predictive performance (expressed by macro-F1 in this paper). This evaluation covers both the single and ensemble versions of these methods. For each dataset, the comparison was performed over different sensor configurations (namely two individual sensors and their fusion) and different numbers of selected features.

The tool condition monitoring was modeled as a five-class classification for our datasets DS1 and DS2, and a three-class classification for DS3 and DS4. We used three classifiers: support vector machine with two tested kernels, namely the quadratic and RBF kernels, as well as the random forest. Based on the experimental results, the conclusions of this paper can be summarized as follows:

- The difference in macro-F1 between different FS methods might be marginal, while the stability difference is quite significant. Such cases promote the role of FS stability as a decisive performance indicator when it comes to selecting the best FS method for a specific monitoring task.
- Given run-to-failure measurements of a milling tool, the sensor configuration, and the number of selected features can affect the FS stability to an extent that depends on the robustness of the FS method itself. Both Fisher score and ReliefF showed a considerably higher consistency (lesser variability) in their stability performance over different settings, compared to mRMR. Further, under the small datasets, the stability of all the FS methods showed a larger dependence on the underlying settings compared to the large datasets.
- Regarding the large datasets of our experiments, DS1 and DS2, the results over the different combinations of sensor configurations, operating conditions, and feature subset cardinalities showed that Fisher score (single and ensemble versions) was the superior FS method in terms of both the stability and predictive performance in almost all the individual cases. Even in the few cases where it was not superior, it showed a high and very comparable performance to the superior one (mostly ReliefF). Thus, Fisher score can be considered a global FS solution for these datasets. As for the small datasets studied in this paper, the results did not suggest a globally superior method, as it varies with the different cases, with the superior one mostly being Fisher score or ReliefF. Further, when a final feature subset cardinality was chosen (at which all the FS methods yielded a reasonable macro-F1) for each dataset/sensor configuration, Fisher score (single and/or ensemble) showed the highest harmonic mean of stability and macro-F1 in most of the cases (24 out of 36 cases). Thus, based on the overall results, we recommend that Fisher score is considered among the top-candidate FS methods to select both stable and predictive features for diagnosis tasks.
- The impact of the homogeneous ensemble generally depends on the FS method, data at hand, and number of selected features. The ensemble technique had overall little impact on Fisher score in the large milling datasets; however, it improves its stability and predictive power in many cases of the small datasets. For all the datasets, mRMR is the method that benefits the most from the ensemble in terms of stability. Especially in the large datasets DS1 and DS2, the mRMR ensemble considerably outperformed the single version in terms of the overall stability and the stability consistency over different settings and in some cases also in the predictive power. This indicates that the ensemble can still be quite beneficial for some FS methods even if the SDR of the data is too high (SDRs for the single sensor/sensor fusion of DS1 and DS2 are 156.06/78.03 and 192.80/96.40, respectively); most of the existing works in other fields focus only on the ensemble benefits with the datasets of SDR<<1. However, it should be noted that there are also cases where the ensemble degrades the stability and/or predictive power. Thus, its trade-off performance should be inspected for the particular case of interest.
- The stability of all the FS methods suffered in some cases under the small datasets and in some cases even after applying the ensemble. This emphasizes the necessity of examining the FS stability and investigating more solutions to increase the FS stability for small datasets since, when it comes to facing the challenges of small datasets, the researches existing in the current PdM literature focus only on how to increase the generalizability of the predictive models.

The Future research directions are as follows:

- The performance comparison presented in this work included only filter-based FS methods, which is a limitation that can be addressed in future work by also evaluating wrappers and embedded methods, e.g., those used in [15,17,24].
- As shown in the results, different factors can affect the stability of a given FS method for PdM applications, e.g., size of dataset, sensors, operating conditions, etc. To gain a more in-depth understanding of the stability behavior with the monitoring data, it is interesting to experimentally determine the most dominating factor among them all. Since the real-world datasets carry the combined effect of the different factors, each factor (e.g., size, operating conditions, etc.) should be changed individually under the same dataset while keeping the other factors constant to isolate their impact (e.g., changing only the size by subsampling the original data with different ratios for the datasets whose size was originally large or creating augmented data and adding them incrementally to the original samples of the datasets whose original size is small) and the resulting variation in stability can be observed.
- Studying the impact of the window size of feature extraction on the joint performance of the FS stability and predictive performance as well as on the ensemble performance in PdM applications.

## Figures and Tables

**Table 1:** The most common performance indicators of the feature selection methods in the existing literature of predictive maintenance.

Performance Indicator of FS	PdM Application	Reference Example(s)
Predictive performance of the model, e.g., accuracy	Tool condition monitoring in milling/micromilling/grinding	[4,35,37]
	Fault diagnosis of gears/bearings/wind turbines/air-handling units/railway point machines	[15,16,18,25,36]
Run time of the FS algorithm	Tool condition monitoring in grinding	[37]
	Fault diagnosis of bearings/wind turbines	[34,18]
Training time of the learning model	Tool condition monitoring in milling	[33]
	Fault diagnosis of bearings	[34]
Number of adjusted parameters in the FS algorithm	Chatter detection in computer numerical control (CNC) machines	[32]
Number of selected features	Fault diagnosis of bearings/railway point machines	[34,36]

**Table 2:** The main characteristics of the filter-based feature selection methods studied in this paper.

FS Method	Metric Type	Evaluation Approach	Feature Redundancy
Fisher score	Statistical	Univariate	Not considered
mRMR	Information-based	Multivariate	Considered
ReliefF	Instance-based	Multivariate	Not considered

**Table 3:** The sensor(s) and main operating conditions of the datasets used in this paper.

Data Set (DS)	Data Source	Sensor(s)	Main Operating Conditions			
			Rotation Speed (rpm)	Feed Rate (mm/min)	Depth of Cut (mm)	Workpiece Material
DS1	Our experiments	Microphone and/or accelerometer	8000	360	2.5	Steel
DS2	Our experiments	Microphone and/or accelerometer	5600	252	2.5	Steel
DS3	NASA’s data repository [26] (case 9)	AC current and/or AE sensor	826	413	1.5	Cast iron
DS4	NASA’s data repository [26] (case 10)	AC current and/or AE sensor	826	206.5	1.5	Cast iron

**Table 4:** The description of the datasets used in this paper.

Data Set	No. of Classes	No. of the Total Observations (per Class 1/…/Class C)	No. of Features (Single Sensor/Sensor Fusion)	SDR ${}^{a}$ (Single Sensor/Sensor Fusion)
DS1	5	12,680 (2247/2599/ 2585/2639/2610)	65/130	156.06/78.03
DS2	5	15,665 (2865/3133/ 3181/3198/3288)	65/130	192.80/96.40
DS3	3	36 (16/8/12)	65/130	0.44/0.22
DS4	3	40 (17/9/14)	65/130	0.49/0.25

${}^{a}$ Sample-to-dimension ratio, computed only on the training observations (80% of the total observations since we use a 5-fold cross-validation).

**Table 5:** The highest macro-F1 value (%) and the corresponding number of selected features across the different datasets and sensor(s). When the maximum macro-F1 for a given FS method is obtained by more than one cardinality of feature subset, only the lowest cardinality is recorded in the table. These results are mainly based on Figure 6 and Figure 7.

Data Set (DS)	FS Method	SVM with a Quadratic Kernel						SVM with an RBF Kernel						Random Forest					
		Max F1	No. Fea	Max F1	No. Fea	Max F1	No. Fea	Max F1	No. Fea	Max F1	No. Fea	Max F1	No. Fea	Max F1	No. Fea	Max F1	No. Fea	Max F1	No. Fea
		Microphone		Accelerometer		Sensor fusion		Microphone		Accelerometer		Sensor fusion		Microphone		Accelerometer		Sensor fusion	
DS1	Fisher	90.47	32	84.74	21	**95.59**	53	**90.54**	34	84.41	21	95.19	36	**91.81**	32	85.35	26	**95.59**	39
	mRMR	90.19	40	83.22	29	94.90	67	89.69	39	82.62	29	94.17	62	**91.81**	36	84.38	30	95.29	50
	ReliefF	**90.59**	33	84.20	24	95.55	42	90.51	33	84.05	27	**95.29**	38	**91.81**	30	**85.39**	26	95.58	36
	No FS	90.26	65	**85.65**	65	94.92	130	89.11	65	**85.35**	65	94.46	130	91.54	65	85.36	65	95.27	130
DS2	Fisher	85.40	33	78.27	15	92.03	51	83.33	33	76.34	15	90.06	47	**83.73**	30	76.96	26	90.07	49
	mRMR	84.86	40	77.16	28	90.94	75	81.86	40	75.75	29	88.71	70	83.71	41	76.98	32	89.51	72
	ReliefF	85.54	32	77.86	17	92.34	36	**83.75**	22	75.94	18	90.51	36	83.56	39	76.72	26	89.92	46
	No FS	**85.89**	65	**78.31**	65	**92.90**	130	83.39	65	**77.36**	65	**91.17**	130	82.67	65	**77.16**	65	**91.07**	130
		AC current		AE sensor		Sensor fusion		AC current		AE sensor		Sensor fusion		AC current		AE sensor		Sensor fusion	
DS3	Fisher	**100**	3	**100**	1	**100**	1	**100**	2	**100**	1	**100**	1	**100**	1	**100**	1	**100**	1
	mRMR	**100**	2	**100**	1	**100**	1	**100**	1	**100**	1	**100**	1	**100**	1	**100**	1	**100**	1
	ReliefF	**100**	3	**100**	1	**100**	3	**100**	3	**100**	1	**100**	3	**100**	1	**100**	1	**100**	1
	No FS	89.33	65	96.44	65	**100**	130	90.22	65	96.44	65	93.33	130	**100**	65	**100**	65	**100**	130
DS4	Fisher	**94.60**	2	91.87	36	89.65	2	**94.60**	2	89.21	1	91.94	2	91.94	29	91.87	27	94.16	24
	mRMR	92.32	1	91.83	1	89.60	1	**94.60**	1	**91.83**	1	92.32	2	**94.60**	17	**94.54**	9	**94.60**	43
	ReliefF	92.32	1	91.87	36	**92.32**	1	**94.60**	1	89.21	1	**94.60**	1	**94.60**	29	91.87	1	**94.60**	2
	No FS	86.38	65	**92.89**	65	83.94	130	88.38	65	89.21	65	85.87	130	**94.60**	65	**94.54**	65	94.54	130

Bold values indicate the FS method with the highest macro-F1 value with respect to the corresponding dataset, classifier, and sensor(s).

**Table 6:** The FS ensemble size (the number of bootstrap samples) that yielded the highest stability among the sizes experimented (10, 20, 30, 40, and 50); if the highest stability is achieved by more than one size, the largest size is recorded. The entries of this table is based on the results shown in Figure 8.

Dataset	Sensor(s)	No. of Selected Features	FS Method	The Best Ensemble Size
DS1	Micro-phone	10/20/30	Fisher	20/50/10
			mRMR	50/50/50
			ReliefF	50/40/50
	Accelero-meter	10/20/30	Fisher	50/50/50
			mRMR	50/50/20
			ReliefF	50/30/50
	Sensor fusion	10/20/30/40/50	Fisher	50/20/30/40/50
			mRMR	40/50/50/40/50
			ReliefF	50/50/40/50/50
DS2	Micro- phone	10/20/30	Fisher	50/50/50
			mRMR	50/40/50
			ReliefF	50/20/20
	Accelero- meter	10/20/30	Fisher	50/50/50
			mRMR	50/50/50
			ReliefF	50/20/50
	Sensor fusion	10/20/30/40/50	Fisher	50/50/50/50/50
			mRMR	50/50/30/50/50
			ReliefF	50/20/50/50/50
DS3	AC current	1/2/3	Fisher	50/30/10
			mRMR	20/40/50
			ReliefF	50/20/40
	AE	1/2/3	Fisher	50/50/50
			mRMR	50/40/40
			ReliefF	50/30/40
	Sensor fusion	1/2/3/4/5	Fisher	50/20/10/50/50
			mRMR	30/30/20/50/50
			ReliefF	50/10/10/20/30
DS4	AC current	1/2/3	Fisher	50/50/50
			mRMR	50/50/50
			ReliefF	50/50/50
	AE	1/2/3	Fisher	50/50/30
			mRMR	50/20/30
			ReliefF	50/20/50
	Sensor fusion	1/2/3/4/5	Fisher	50/30/20/30/30
			mRMR	50/30/30/30/30
			ReliefF	50/50/20/20/40

**Table 7:** Macro-F1 and stability results of the studied FS methods (single and ensemble versions) for the datasets DS1 and DS2. The ensemble sizes are as defined in Table 6.

Sensor(s)	No. of Fea	FS Method	Dataset DS1								Dataset DS2							
			Stability (%)		Macro-F1 (%)						Stability (%)		Macro-F1 (%)					
					SVM-poly2		SVM-RBF		RF				SVM-poly2		SVM-RBF		RF	
			Sing	Ens	Sing	Ens	Sing	Ens	Sing	Ens	Sing	Ens	Sing	Ens	Sing	Ens	Sing	Ens
Micro- phone	10	Fisher	89.85	**91.30**	83.69	83.53	85.10	84.84	**89.37**	**89.49**	**100**	**100**	**77.95**	**77.95**	**78.74**	**78.74**	**79.09**	**78.65**
		mRMR	68.09	84.04	82.03	82.47	82.57	83.15	88.34	88.53	66.84	84.14	72.61	71.35	72.37	70.17	75.40	72.65
		ReliefF	**94.20**	85.49	**84.65**	**84.47**	**85.57**	**85.12**	88.81	88.85	89.91	94.23	72.40	70.74	71.21	69.34	70.82	68.55
	20	Fisher	**92.34**	**94.26**	**88.86**	**88.63**	**89.49**	**89.62**	**91.19**	91.17	**100**	**100**	**83.73**	**83.71**	81.95	**81.96**	**82.31**	**81.86**
		mRMR	67.49	90.44	86.81	86.97	86.95	87.11	90.82	**91.22**	71.48	81.95	80.40	80.48	79.04	79.65	81.35	81.65
		ReliefF	84.71	88.54	88.65	88.46	89.12	88.87	90.66	90.77	93.34	91.44	83.15	82.11	**83.18**	81.63	81.96	81.64
	30	Fisher	**93.27**	**94.95**	90.10	**90.31**	90.15	90.14	91.30	**91.80**	90.78	90.78	**85.18**	**85.18**	83.21	**83.21**	**83.73**	**83.60**
		mRMR	82.33	78.12	89.15	88.73	89.00	88.47	91.61	91.48	74.87	84.08	83.56	83.85	81.55	81.66	82.96	82.56
		ReliefF	89.91	92.43	**90.43**	90.11	**90.26**	**90.55**	**91.81**	91.46	**91.62**	**100**	85.07	82.66	**83.63**	80.92	83.46	82.80
Accelero- meter	10	Fisher	**100**	**100**	83.84	83.84	83.81	83.81	84.46	84.45	**100**	**100**	**77.39**	**77.40**	**75.87**	**75.87**	**75.92**	**76.16**
		mRMR	71.07	93.92	81.06	76.23	79.53	74.61	82.12	76.59	60.97	83.47	64.68	73.20	61.66	71.50	64.49	72.70
		ReliefF	**100**	**100**	**84.01**	**84.07**	**83.95**	**84.02**	**84.71**	**84.72**	81.99	90.99	76.34	73.98	73.44	71.86	74.26	72.84
	20	Fisher	**100**	**100**	**84.53**	**84.53**	**83.79**	**83.79**	84.81	**84.70**	**100**	**100**	**77.98**	**77.98**	**76.16**	**76.16**	**76.79**	**76.75**
		mRMR	66.70	88.55	82.67	82.79	81.97	82.31	83.83	83.97	65.54	82.76	76.43	75.36	75.27	73.45	76.24	74.29
		ReliefF	89.59	81.27	83.87	83.60	83.65	83.30	**85.05**	84.55	82.77	85.81	77.65	76.88	75.76	75.04	76.42	75.82
	30	Fisher	**96.27**	**96.27**	**84.02**	**84.02**	83.60	83.60	85.02	**85.14**	**100**	**96.40**	**77.11**	**77.20**	75.67	75.60	76.34	76.64
		mRMR	86.04	86.05	83.11	83.13	82.50	82.45	84.38	84.05	79.27	89.18	76.96	76.85	**75.68**	**75.74**	**76.91**	**76.95**
		ReliefF	94.42	94.42	83.76	83.77	**83.97**	**83.78**	**85.06**	85.08	87.39	**96.40**	77.04	76.80	75.52	75.07	76.44	76.30
Sensor fusion	10	Fisher	**100**	**100**	**88.83**	**88.83**	**89.84**	**89.84**	**93.40**	**93.50**	**100**	**100**	**86.77**	**86.77**	**86.72**	**86.72**	**86.54**	**86.48**
		mRMR	51.06	77.41	85.39	79.19	85.33	78.80	88.61	85.05	58.95	77.62	62.02	78.75	59.55	78.47	62.13	79.40
		ReliefF	**100**	**100**	88.07	88.07	89.50	89.50	93.04	93.23	90.05	95.02	79.13	78.87	78.23	77.88	78.37	78.25
	20	Fisher	**94.76**	**95.51**	**93.38**	**93.63**	**93.52**	**93.63**	**94.63**	**94.73**	**97.05**	**97.05**	**90.83**	**90.84**	**89.70**	**89.70**	**88.67**	**88.77**
		mRMR	72.30	85.77	89.88	89.01	89.79	88.58	92.44	93.22	58.69	84.50	80.76	82.12	79.59	81.17	83.06	81.71
		ReliefF	91.77	90.27	92.57	92.36	93.01	92.62	94.30	94.46	91.88	**97.05**	85.18	88.43	84.12	87.08	83.70	86.02
	30	Fisher	**97.66**	**100**	**94.90**	**94.82**	**94.76**	**94.77**	**95.13**	**95.10**	**100**	**100**	91.85	**91.85**	89.89	**89.91**	89.31	**88.93**
		mRMR	67.23	91.22	92.90	92.72	92.19	92.65	94.50	94.71	69.01	86.24	88.29	83.09	86.88	82.40	88.23	82.40
		ReliefF	95.32	94.15	94.85	94.56	94.62	94.57	94.87	94.94	94.84	91.40	**91.93**	90.25	**90.45**	88.40	**89.52**	87.77
	40	Fisher	**95.43**	**95.94**	95.46	**95.42**	95.12	**95.11**	**95.42**	**95.46**	**100**	**100**	91.88	**91.88**	89.69	**89.69**	**89.58**	**89.34**
		mRMR	81.20	88.31	94.16	93.36	93.76	93.04	94.98	94.73	67.25	86.61	89.14	85.68	87.69	84.55	88.64	84.85
		ReliefF	86.77	87.29	**95.47**	95.21	**95.16**	94.91	**95.42**	95.37	92.56	88.59	**92.23**	90.80	**90.25**	89.01	89.36	88.85
	50	Fisher	**98.12**	**96.72**	**95.39**	**95.41**	94.85	**94.87**	95.49	95.50	**94.05**	**94.05**	91.86	**91.86**	**89.97**	**89.97**	**90.03**	**89.98**
		mRMR	84.06	86.88	94.69	93.54	94.07	93.24	95.29	94.88	71.64	85.37	89.82	89.79	88.41	88.27	88.96	88.90
		ReliefF	87.34	93.90	95.38	95.36	**94.97**	94.84	**95.52**	**95.51**	90.85	93.14	**92.05**	90.61	89.88	88.76	89.80	88.90

Bold values indicate the best feature selection (FS) method for the corresponding case and metric. Underlined values indicate which of the single and ensemble versions of a specific FS is better.

**Table 8:** Macro-F1 and stability results of the studied FS methods (single and ensemble versions) for the datasets DS3 and DS4. The ensemble sizes are as defined in Table 6.

Sensor(s)	No. of Fea	FS Method	Dataset DS3								Dataset DS4							
			Stability (%)		Macro-F1 (%)						Stability (%)		Macro-F1 (%)					
					SVM-poly2		SVM-RBF		RF				SVM-poly2		SVM-RBF		RF	
			Sing	Ens	Sing	Ens	Sing	Ens	Sing	Ens	Sing	Ens	Sing	Ens	Sing	Ens	Sing	Ens
AC current	1	Fisher	**100**	**100**	**94.44**	**94.44**	**96.83**	**96.83**	**100**	**100**	**100**	**100**	91.64	91.64	91.64	91.64	85.21	88.38
		mRMR	**100**	35.49	**94.44**	83.64	**100**	82.91	**100**	74.75	**100**	35.58	**92.32**	64.60	**94.60**	62.34	**88.98**	81.43
		ReliefF	**100**	**100**	**94.44**	**94.44**	**96.83**	**96.83**	**100**	**100**	**100**	**100**	**92.32**	**92.32**	**94.60**	**94.60**	**88.98**	**88.98**
	2	Fisher	77.14	**100**	96.44	**100**	**100**	**100**	**100**	**100**	**77.25**	60.19	**94.60**	**92.32**	**94.60**	**94.60**	88.38	88.38
		mRMR	**100**	60.08	**100**	**100**	**100**	**100**	**100**	94.22	54.47	**60.25**	89.14	91.05	**94.60**	91.05	**88.98**	**91.05**
		ReliefF	54.35	65.79	94.44	96.44	96.83	**100**	**100**	**100**	60.23	60.23	91.05	91.05	**94.60**	**94.60**	**88.98**	88.98
	3	Fisher	**83.97**	**100**	**100**	**100**	**100**	**100**	**100**	**100**	**68.10**	**68.10**	86.38	89.05	89.05	89.05	82.83	85.49
		mRMR	59.95	75.97	86.67	**100**	89.33	**100**	**100**	91.05	56.14	48.20	82.98	82.10	88.76	87.87	**88.98**	**91.05**
		ReliefF	71.97	83.97	**100**	**100**	**100**	**100**	**100**	**100**	64.14	64.14	**91.05**	**91.05**	**91.05**	**91.05**	88.16	88.16
AE	1	Fisher	**100**	**100**	**100**	**100**	**100**	**100**	**100**	**100**	**100**	**100**	86.25	86.25	89.21	**89.21**	88.70	88.70
		mRMR	**100**	**100**	**100**	68.44	**100**	53.27	**100**	64.44	77.71	55.13	**91.83**	66.76	**91.83**	72.87	85.97	67.48
		ReliefF	**100**	**100**	**100**	**100**	**100**	**100**	**100**	**100**	**100**	**100**	89.21	**89.21**	89.21	**89.21**	**91.87**	**91.87**
	2	Fisher	64.46	64.46	96.44	**96.44**	96.44	**96.44**	**100**	**100**	58.24	**75.99**	**87.05**	82.54	**87.05**	**89.21**	88.70	88.70
		mRMR	46.76	**100**	**100**	67.43	**97.33**	64.48	**100**	68.44	28.08	57.91	78.25	70.98	76.57	75.49	88.19	78.19
		ReliefF	**76.33**	76.33	89.78	89.78	92.89	92.89	**100**	**100**	**100**	64.14	**87.05**	**87.56**	**87.05**	87.94	**91.87**	**91.87**
	3	Fisher	**58.33**	58.33	**86.22**	86.22	89.78	**89.78**	**96.44**	**96.44**	45.13	49.38	**80.16**	80.16	**89.21**	86.32	86.25	86.25
		mRMR	24.97	**66.64**	84.00	**86.54**	**91.11**	77.65	91.11	89.33	28.07	45.05	78.35	65.65	79.08	68.70	88.19	74.48
		ReliefF	**58.33**	58.34	**86.22**	86.22	89.78	**89.78**	**96.44**	**96.44**	**70.36**	**57.97**	77.27	**83.94**	**89.21**	**89.21**	**89.21**	**89.21**
Sensor fusion	1	Fisher	**100**	**100**	**100**	**100**	**100**	**100**	**100**	**100**	89.44	**89.44**	84.03	84.03	86.98	**86.98**	86.48	86.48
		mRMR	**100**	3.95	**100**	85.33	**100**	68.54	**100**	64.76	57.50	25.54	89.60	38.89	89.60	36.81	**92.32**	54.49
		ReliefF	**100**	**100**	94.44	94.44	96.83	96.83	**100**	**100**	**100**	46.89	**92.32**	**86.98**	**94.60**	**86.98**	88.98	**89.65**
	2	Fisher	**60.76**	**77.54**	**96.44**	**96.44**	**100**	**100**	**100**	**100**	61.09	61.14	89.65	86.98	91.94	89.27	86.48	86.48
		mRMR	49.55	32.69	93.78	92.89	93.78	92.89	**100**	87.56	33.35	38.79	86.43	68.92	92.32	81.08	92.32	83.98
		ReliefF	60.75	66.34	94.44	**96.44**	96.83	**100**	**100**	**100**	**100**	**77.78**	**92.16**	**87.33**	**94.54**	**92.38**	**94.60**	**89.65**
	3	Fisher	**76.67**	76.68	**100**	**100**	**100**	**100**	97.33	**97.33**	46.11	46.17	86.98	85.56	**89.65**	91.49	89.27	89.27
		mRMR	53.37	37.83	92.89	92.89	93.78	92.89	**100**	81.21	30.59	57.64	80.19	75.89	82.63	91.87	83.43	86.98
		ReliefF	68.85	**84.42**	**100**	**100**	**100**	**100**	**100**	**97.33**	**73.02**	**76.90**	**89.49**	**86.83**	89.49	**94.54**	**89.65**	**89.65**
	4	Fisher	**81.93**	**81.93**	**100**	**100**	**100**	**100**	**100**	**100**	43.34	49.35	86.98	86.83	**89.49**	**91.87**	86.98	89.27
		mRMR	45.76	54.82	93.78	92.89	91.11	92.89	96.44	87.49	16.43	67.12	78.76	91.78	80.87	88.98	84.05	**91.94**
		ReliefF	60.82	66.87	96.44	96.44	**100**	**100**	**100**	**100**	**61.18**	**67.17**	**89.21**	**91.87**	89.21	**91.87**	**89.21**	89.21
	5	Fisher	67.76	67.76	92.89	92.89	92.89	92.89	**100**	**100**	43.49	50.91	86.83	86.83	86.83	89.21	86.98	89.27
		mRMR	33.07	65.31	88.44	**100**	88.44	**96.83**	91.11	93.65	16.52	50.81	78.98	82.60	81.14	83.43	84.32	89.27
		ReliefF	**75.21**	**67.77**	**100**	96.44	**100**	96.44	97.33	**100**	**63.19**	**75.44**	**89.21**	**89.21**	**89.21**	**91.87**	**89.21**	**91.49**

Bold values indicate the best feature selection (FS) method for the corresponding case and metric. Underlined values indicate which of the single and ensemble versions of a specific FS is better.

**Table 9:** Harmonic mean (HM) of stability and macro-F1 for the FS methods (single and ensemble versions) across the datasets and sensor(s).

Sensor(s)	No. of Selected Features	Feature Selection Method	Dataset DS1						Dataset DS2					
			HM (%)						HM (%)					
			SVM-poly2		SVM-RBF		RF		SVM-poly2		SVM-RBF		RF	
			Sing	Ens	Sing	Ens	Sing	Ens	Sing	Ens	Sing	Ens	Sing	Ens
Micro- phone	30	Fisher	91.66	**92.57**	91.68	**92.48**	92.27	**93.35**	87.89	87.89	86.83	86.83	87.11	87.04
		mRMR	85.60	83.09	85.54	82.97	86.72	84.27	78.98	83.96	78.07	82.85	78.70	83.31
		ReliefF	90.17	91.26	90.08	91.48	90.85	91.94	88.22	**90.51**	87.44	**89.45**	87.35	**90.59**
Accelero- meter	20	Fisher	**91.62**	**91.62**	**91.18**	**91.18**	**91.78**	91.72	**87.63**	**87.63**	**86.47**	**86.47**	**86.87**	86.85
		mRMR	73.83	85.57	73.55	85.32	74.29	86.20	70.57	78.89	70.07	77.83	70.49	78.30
		ReliefF	86.64	82.42	86.52	82.27	87.26	82.88	80.13	81.10	79.11	80.06	79.47	80.51
Sensor fusion	40	Fisher	95.44	**95.68**	95.27	**95.52**	95.43	**95.70**	**95.77**	**95.77**	**94.56**	**94.56**	**94.50**	94.37
		mRMR	87.20	90.76	87.03	90.61	87.55	91.41	76.66	86.14	76.12	85.57	76.48	85.72
		ReliefF	90.91	91.08	90.77	90.94	90.89	91.15	92.39	89.68	91.39	88.80	90.93	88.72
			Dataset DS3						Dataset DS4					
AC current	2	Fisher	85.72	**100**	87.10	**100**	87.10	**100**	**85.05**	72.87	**85.05**	73.57	**82.44**	71.61
		mRMR	**100**	75.06	**100**	75.06	**100**	73.37	67.62	72.52	69.13	72.52	67.57	72.52
		ReliefF	68.99	78.22	69.62	79.37	70.42	79.37	72.50	72.50	73.60	73.60	71.84	71.84
AE	2	Fisher	77.27	77.27	77.27	77.27	78.39	78.39	69.79	79.13	69.79	82.07	70.31	81.85
		mRMR	63.72	80.55	63.17	78.40	63.72	81.26	41.33	63.78	41.09	65.54	42.60	66.54
		ReliefF	**82.51**	**82.51**	**83.80**	**83.80**	**86.58**	**86.58**	**93.08**	74.04	**93.08**	74.18	**95.76**	75.54
Sensor fusion	2	Fisher	74.55	**85.96**	75.59	**87.35**	75.59	**87.35**	72.66	71.81	73.41	72.57	71.60	71.64
		mRMR	64.84	48.36	64.84	48.36	66.27	47.61	48.13	49.64	49.00	52.48	49.00	53.07
		ReliefF	73.94	78.61	74.66	79.76	75.58	79.76	**95.92**	82.28	**97.19**	84.45	**97.23**	83.29

Bold values indicate the best feature selection (FS), among all the single and ensemble versions, for the corresponding dataset, sensor(s), and classifier. Underlined values indicate which of the single and ensemble versions of a specific FS is better for the corresponding case.

## Data Availability

The first two datasets used in this paper are available upon request from the corresponding author. The last two datasets are publicly available (See Reference [26]).

## Abbreviations

The following abbreviations are used in this manuscript:

CNC	Computer Numerical Control
DAS	Data Acquisition System
ECG	Electrocardiogram
FN	False Negative
FP	False Positive
FS	Feature Selection
HM	Harmonic Mean
MIQ	Mutual Information Quotient
mRMR	Minimum Redundancy Maximum Relevance
PdM	Predictive Maintenance
RF	Random Forest
RMS	Root Mean Square
SDR	Sample-to-Dimension Ratio
STFT	Short-Time Fourier Transform
SVM	Support Vector Machine
TCM	Tool Condition Monitoring
TN	True Negative
TP	True Positive
